# The link to steady-state oxidative metabolism and hemodynamics varies across rs-fMRI metrics: A whole-brain assessment using macrovascular correction

**DOI:** 10.1162/IMAG.a.1060

**Published:** 2025-12-11

**Authors:** Xiaole Z. Zhong, Hannah Van Lankveld, J. Jean Chen

**Affiliations:** Rotman Research Institute at Baycrest, Toronto, ON, Canada; Department of Medical Biophysics, University of Toronto, Toronto, ON, Canada; Department of Biomedical Engineering, University of Toronto, Toronto, ON, Canada

## Abstract

One of the major obstacles to the clinical application of resting-state functional magnetic resonance imaging (rs-fMRI) is the complex nature of its measurements, which limits interpretability. An approach to enhance the interpretability of the rs-fMRI metrics is to link them to more fundamental brain physiology, especially cerebral metabolism. Previous studies have established associations between glucose metabolism (CMR_glu_) and rs-fMRI measurements. In spite of this, oxidative metabolism (CMRO_2_) is more closely related to cerebral blood flow (CBF) and thus the blood-oxygenation level-dependent (BOLD) signal, and its relationship with CMR_glu_ is complex. Additionally, most currently published rs-fMRI metrics are uncorrected for macrovascular contribution, which may obscure the neuronal contributions. In this study, we measured resting CMRO_2_ (along with the oxygen extraction fraction, OEF, and cerebral blood flow, CBF) using gas-free calibrated fMRI. We used linear mixed-effects (LME) models to examine associations between CMRO_2_ and various rs-fMRI metrics before and after macrovascular correction. We found that (1) significant associations existed between CMRO_2_ and multiple rs-fMRI metrics, with the strongest association found for the global functional density (gFCD) and the weakest for seed-based functional connectivity (FC); (2) associations with rs-fMRI metrics also varied for OEF and CBF; (3) significant sex differences were observed in the above associations; (4) the use of macrovascular correction substantially strengthened the goodness fit of all LME models examined. The latter improvement further validates the use of macrovascular correction in rs-fMRI. These results provide a framework for linking rs-fMRI metrics to fundamental brain physiology, thus improving interpretability of rs-fMRI measurements. This is the first study to formally link whole-brain MRI-based baseline CMRO_2_ and rs-fMRI metrics, and helps to push the envelope for rs-fMRI in future clinical applications.

## 1 Introduction

Resting-state functional magnetic resonance imaging (rs-fMRI), most commonly based on the blood-oxygenation level-dependent (BOLD) contrast, aims to use spontaneous brain activity (B. Biswal et al., 1995) to investigate brain function in health and disease. Most rs-fMRI studies lend themselves to connectivity analysis, originally based on Pearson’s correlation (B. Biswal et al., 1995) and later developed into a family of connectivity metrics. Moreover, the resting-state fluctuation amplitude (RSFA) has been used as a means of understanding the temporal variability of brain-related signals underlying connectivity measures (Kannurpatti et al., 2011). Recently, non-linear metrics, notably entropy, have revealed novel insight into the relationship between the rs-fMRI signal complexity and cognitive performance (Wang, 2021). In spite of the numerous metrics that have been proposed as markers of neuronal function, there has been limited application in clinical practice, due in part to the lack of understanding of the neuronal mechanism that underlies the rs-fMRI signal (Lee et al., 2013). For instance, many of these metrics have significant vascular contributions that may obscure the metabolic interpretations (vice versa).

A natural approach to investigating this question of neuronal mechanisms of rs-fMRI has been to relate the fMRI signal to tasks. Local neuronal specificity has been investigated by linking fMRI to its neurovascular substrates, including cerebral blood flow (CBF) and cerebral metabolic rate of oxygen (CMRO_2_), which are believed to be more directly associated with local neural activity (Davis et al., 1998). In the task literature, typically, a reduction in OEF caused by an increase in CBF in response to a CMRO_2_ increase is associated with an increase in BOLD fMRI during local neural activation. Such associations are also relevant to understanding rs-fMRI if the same dynamic coupling for tasks is assumed to apply at rest. This has led to previous research suggesting that functional networks can be derived not only from fluctuations in rs-fMRI but also from the fluctuation of CMRO_2_ and CBF (B. B. Biswal et al., 1997; Wu et al., 2009). However, this approach assumes a dynamic neurovascular coupling similar to a task-related steady-state coupling, which has not been established. Treating resting state as a form of steady state, an association between RSFA and static CBF by simulations and in vivo measurements has been reported (Chu et al., 2018). Nevertheless, the relationship between rs-fMRI connectivity and steady-state resting CMRO_2_ has not yet been fully established. While this relationship is understandably distinct from that in task or dynamic state, it is nevertheless an important step in understanding and interpreting rs-fMRI metrics.

A seminal study by Raichle et al. (2021) using positron emission tomography (PET) demonstrated that baseline CMRO_2_ is distributed heterogeneously across the resting brain, being particularly high in a set of functionally connected regions constituting the default-mode network (DMN). In light of the high resting metabolism of the DMN regions, it is likely that it is the most active in the resting state. This evidence encourages the investigation of neurometabolism in other resting-state brain networks. Although such investigations have been limited, the cerebral metabolic rate of glucose (CMR_glu_) has also been used to establish links between rs-fMRI and brain metabolism at rest. As an example, a positive association has been established between CMR_glu_ and functional connectivity density (FCD) across different brain regions (Shokri-Kojori et al., 2019; Tomasi et al., 2013), where hub regions, which were highly connected locally and globally, were associated with higher CMR_glu_ (Tomasi & Volkow, 2010). The above research into differences in BOLD-metabolic coupling based on network-wise differences motivated our network-driven approach (Raichle et al., 2001; Tomasi et al., 2013), which also provides the possibility of extending the findings to previous or future non-MRI-based measures (e.g., electroencephalography (Phadikar et al., 2025; Schimmelpfennig et al., 2023; X. Zhong et al., 2025) or PET (Hahn et al., 2024; Scherr et al., 2021; M. Zhang et al., 2022)).

CMR_glu_, however, is not directly related to the rs-fMRI signal, which is based on the BOLD effect and thus more directly related to oxygenation and thus CMRO_2_ (Davis et al., 1998; Hoge et al., 1999; Ogawa et al., 1993). In spite of the fact that the majority of glucose is thought to undergo complete oxidative phosphorylation, the metabolism of glucose and oxygen is not always coupled (Gibbs et al., 1942). Moreover, glycolysis can occur not only through a lack of oxygen but also as a failure to fully metabolize glucose in spite of having sufficient oxygen (under-consumption of oxygen), a scenario that is also found in the healthy brain, with the highest incidence located within the DMN (Vaishnavi et al., 2010). The above sources of decoupling between CMRO_2_ and CMRglu highlight the importance of the initial study of CMRO_2_ by Raichle et al. (2001), which, however, did not explicitly involve rs-fMRI metrics.

Establishing a connection between rs-fMRI and metabolism would require that rs-fMRI metrics be dominated by neuronal sources. However, the BOLD signal can be significantly affected by physiological factors that are not localized to neuronal activity, which makes it more difficult to interpret the rs-fMRI metrics. The typical frequency range of neural activity fluctuations in rs-fMRI is 0.01 to 0.1 Hz (Tong, Hocke, et al., 2019), though time-lag spatiotemporal correlational structures are also observed among low-frequency oscillations (sLFOs) within this frequency range (Tong et al., 2015). It is also found that the sLFOs are especially prominent around the macrovasculature, especially large veins (X. Z. Zhong, Tong, et al., 2024), contributing to robust non-neural correlation patterns (Tong, Yao, et al., 2019). Even in the early days of fMRI, the macrovasculature (defined as major arteries and sinuses) was found to have a greater influence on BOLD signal than microvasculature (defined as capillaries, arterioles, and venules) (Boxerman et al., 1995). The same is true for higher fields (Menon, 2012), even as the sensitivity to microvasculature increases (Menon & Goodyear, 1999). In fact, rs-fMRI has been demonstrated in direct extraction of venous structure in rats at 9.4 T (Hyde & Li, 2014), and at 11.7 T, the venous BOLD response is twice as high as that in brain tissue (Yu et al., 2012). As systematically assessed in our recent study (X. Z. Zhong, Tong, et al., 2024), the macrovasculature consistently exhibited high correlations within itself and that these correlations are responsible for a considerable portion of apparent across gray matter regions (can be up to ~30% of voxel-specific total gray matter connectivity), especially as the contribution extends well beyond the boundaries of macrovasculature. The signal contribution to the perivascular tissue can extend as much as 1.2 cm from macrovascular boundaries, which increases the difficulty of correcting macrovascular bias. Thus, the macrovascular confound is non-trivial, and its effect on the neuronal interpretation of rs-fMRI is a profound unknown. This may have hampered the effort in understanding the neuronal basis of rs-fMRI metrics. Recently, we proposed a novel method to correct for such macrovascular effects using biophysical modelling (X. Z. Zhong, Polimeni, et al., 2024), but its effect on the interpretability of rs-fMRI metrics has yet to be demonstrated. We think that the link to baseline physiological metrics such as CMRO_2_ can help establish such an interpretation, as described earlier.

In this study, we extend previous studies that examined how network-based rs-fMRI metrics (RSFA, gFCD, and lFCD) (Shokri-Kojori et al., 2019; Tomasi et al., 2013) were associated with glycolytic metabolism, and focus on how they are associated with oxidative metabolism. We continue the well-regarded assumption that a higher degree of connectivity would require a higher level of metabolic supply. In order to provide a more comprehensive understanding of the rs-fMRI as a whole, we extended our analysis by including two other metrics: seed-based FC, which is a common and basic method of network analysis, and fMRI entropy, which is a novel nonlinear metric that may be able to capture BOLD signal features that linear metrics (RSFA and connectivity) fail to capture. We hypothesize that these different rs-fMRI metrics are all associated with CMRO_2_, but to varying degrees due to their different neuronal specificity. Specifically, rs-fMRI metrics that have a higher neuronal specificity are expected to be more strongly associated with CMRO_2_, which is used as a surrogate for local neural activity, and the two imaging measurements that are acquired to estimate CMRO_2_ (namely, OEF and CBF). We further hypothesize that with macrovascular correction, rs-fMRI metrics can become better explained by the CMRO_2_. This would be an important step towards understanding the extent to which macrovascular BOLD is related or unrelated to neuronal activity, and could provide a solid basis for the interpretation of rs-fMRI findings and the selection of metrics or analysis pipelines in future work.

## 2 Methods

### 2.1 Theory: gas-free calibrated fMRI

In this study, the metabolic metric CMRO_2_ was calculated using gas-free calibrated fMRI, in which OEF is estimated based on the Yablonskiy random-cylinder model (Hirsch et al., 2014; Yablonskiy & Haacke, 1994). The OEF was estimated based on

$$OEF=\frac{R_{2}^{'}}{\frac{4}{3}\pi \times \gamma \times \Delta \gamma \times B_{0}\times CBV},$$ (1)

where *R*_2′_ is estimated from *R*_2_ and *R*_2_* as will be described in the subsection titled *Baseline metabolism estimation*, and reflects MR signal dephasing resulting from local magnetic field inhomogeneities. γ is the proton gyromagnetic ratio, and ∆χ is the susceptibility difference between oxygenated and deoxygenated blood, CBV is cerebral blood volume, and its estimation will be described in the *Baseline perfusion estimation* section. OEF was restricted to be between 0 and 1. Using the OEF estimate, quantitative CMRO_2_ can then be estimated (Göttler et al., 2019) as follows:

$$CMRO_{2}=CaO_{2}\times CBF\times OEF,$$ (2)

where CaO_2_ is the arterial oxygen content, set to $19\;{\text{ml O}}_{2}/100\;\text{ml blood}$ (An et al., 2001).

### 2.2 Participants

This study involves 20 young healthy participants (10 M/10 F, age = 20 to 32 years). No participant reported having a history of cardiovascular disease, psychiatric illness, neurological disorder, malignant disease, or medication use that may have affected the study. Participants were recruited through the Baycrest Participants Database. The study was approved by the research ethics board (REB) of Baycrest, and the experiments were performed with written consent of each participant according to REB guidelines.

### 2.3 MRI acquisition

The images were acquired using a Siemens Prisma 3 Tesla System (Siemens, Erlangen, Germany), which employed 20-channel phased-array head coil reception and body coil transmission. The following data were acquired for each participant: (i) T1-weighted structural image (sagittal, 234 slices, 0.7 mm isotropic resolution, TE = 2.9 ms, TR = 2240 ms, TI = 1130 ms, flip angle = 10^o^); (ii) two time-of-flight (TOF) scans, with coronal and sagittal flow encoding, respectively (coronal: 0.8 x 0.8 x 2.125 mm thickness, 100 slices, TR = 16.6 ms, TE = 5.1 ms, flip angle (α) = 60^o^; sagittal: 0.8 x 0.8 x 2.125 mm thickness, 80 slices, TR = 16.6 ms, TE = 5.1 ms; flip angle (α) = 60^o^); (iii) one dual-echo (DE) pseudo-continuous arterial spin labelling (pCASL) (courtesy of Danny J. J. Wang, University of Southern California) for recording CBF and BOLD dynamics (TR = 4.5 s, TE1 = 9.8 ms, TE2 = 30 ms, post-labelling delay = 1.5 s, labelling duration = 1.5 s, flip angle (α) = 90^o^, 3.5 mm isotropic resolution, 35 slices, slices gap = 25%, scanning time = 4 min), complete with an M_0_ scan in which the TR was 10 s and the scan time 45 s, all other parameters remaining the same; (iv) one multi-echo gradient echo (MEGRE) scan for static R_2_^*^ mapping (TR = 4 s, TE ranged from 5 ms to 60 ms with the step of 5 ms, flip angle = 20^o^, magnitude and phase recording, same spatial resolution as the pCASL); (v) SE MRI data for static R_2_ mapping (TR = 10 s, TE = [17, 33, 50, 66, 91, 107, 116] ms, same spatial resolution as the pCASL). The participants were imaged while viewing naturalistic stimuli to minimize random mind wandering, thereby enhancing the reproducibility of the results (Gal et al., 2022).

### 2.4 Macrovascular correction

An overview of the BOLD signal simulation framework can be found in our previous study (X. Z. Zhong, Polimeni, et al., 2024). The macro-VAN (vascular anatomical network) model obtained from the TOF data provided a more accurate estimation of macrovascular BOLD signals, particularly from venous vessels.

#### 2.4.1 Blood vessel segmentation

**Fig. 1.**
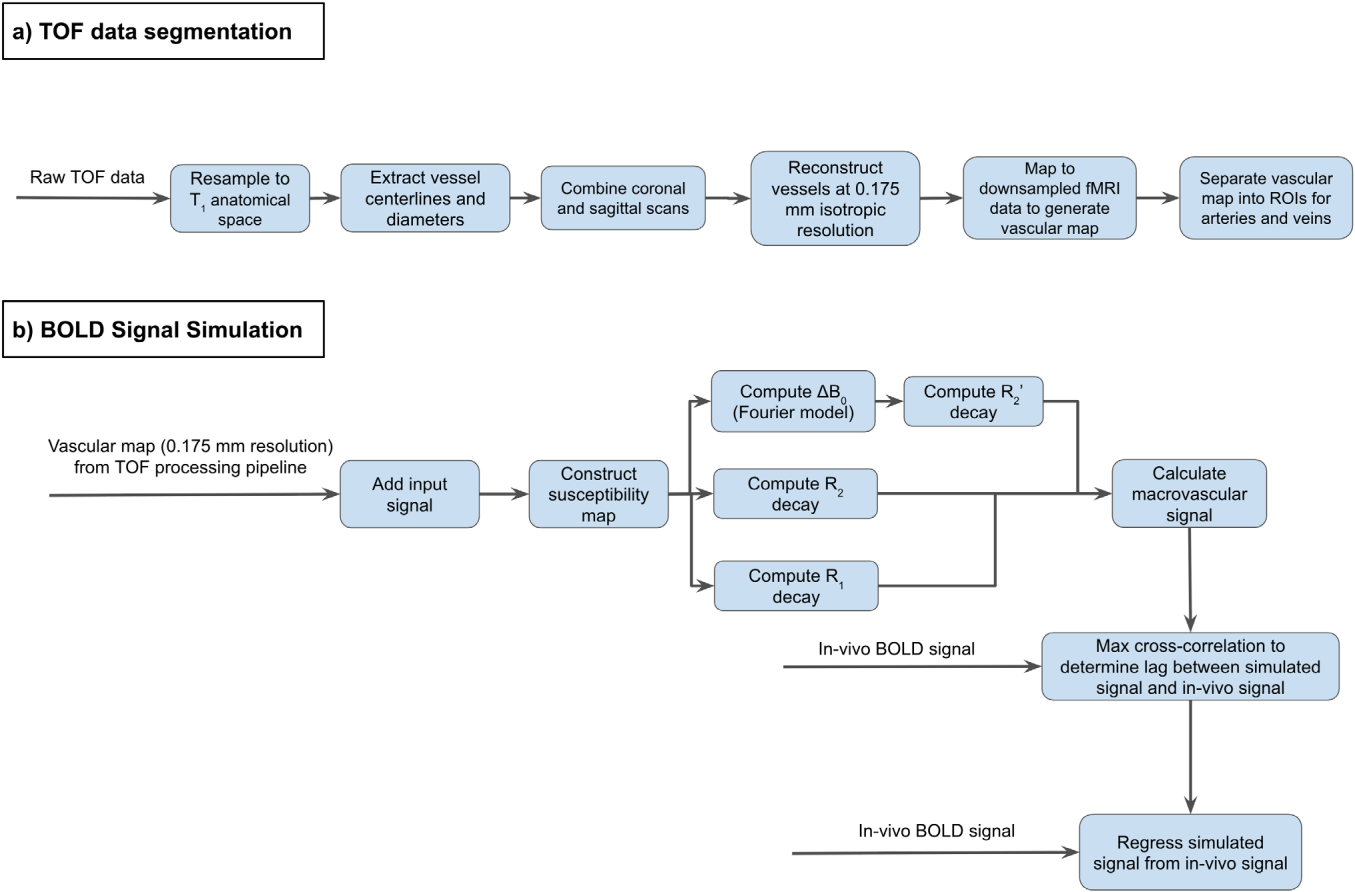
Overview of the macrovascular correction procedure. (a) The TOF data preprocessing and segmentation pipeline; (b) BOLD signal simulation and regression.

**Fig. 2.**
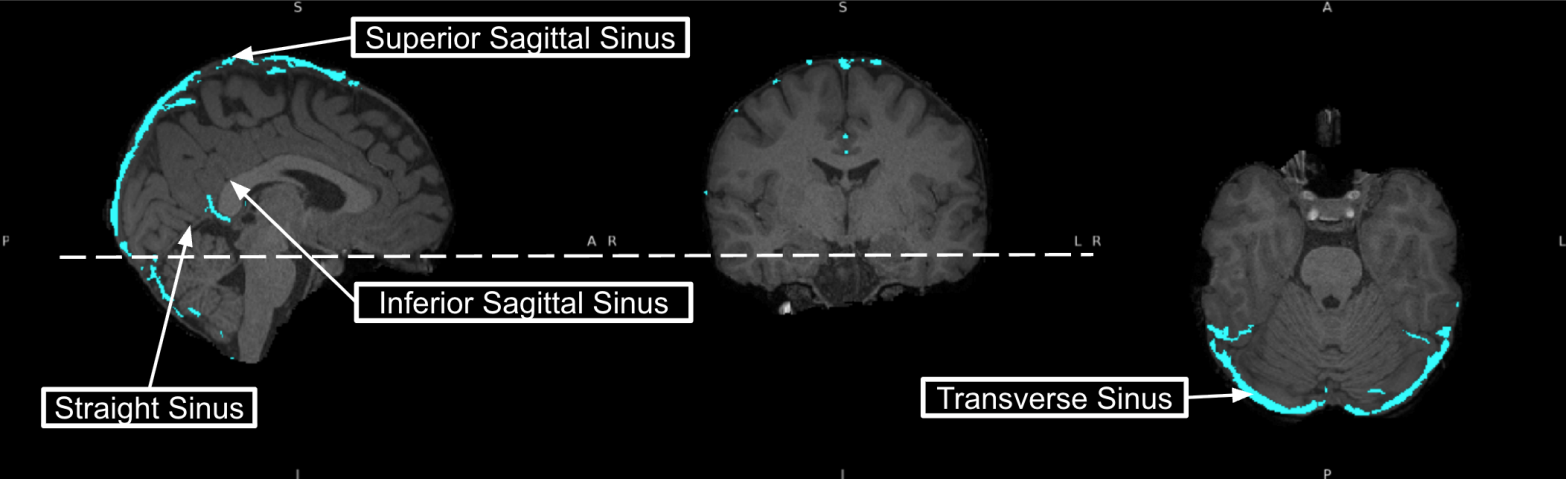
Venous vasculature demonstration overlays the anatomical images and defines macrovasculature in this study (data shown from a representative data set). Blue is the vein segmented from the TOF data overlay on the T1 anatomical image; the white dashed line indicates the axial slice position.

The first step for macrovascular correction is the creation of a macro-VAN, or vascular mask. The strategies for segmenting and processing macrovasculature are summarized in Figure 1a. For each participant, TOF data were registered to T1 space (FSL MCFLIRT, DOF = 6, cost function = correlation) and then segmented using the BrainCharter Toolbox (Bernier et al., 2018). Visual inspection was conducted to ensure that the macrovasculature segmentations were free of artefacts (e.g., wrap-around artefacts). The vascular segmentations from both encoding directions were summed and then binarized to produce the final macro-VAN segmentation. The final segmentations were first upsampled to a 0.175 mm (i.e., 0.7 mm/4) isotropic resolution (required for the simulation; see our previous work X. Z. Zhong, Polimeni, et al., 2024) and then downsampled to the rs-fMRI resolution (3.5 mm resolution). The macrovascular blood-volume fraction (fBV) was estimated by counting the number of high-resolution voxels (0.175 mm isotropic resolution) occupied by vessels in each rs-fMRI voxel (3.5 mm isotropic resolution with a 25% slice gap). As the TOF data contained arteries, veins, and false-positive detections, they had to be manually identified and separated into separate maps using the anatomical atlas (Tortora & Derrickson, 2018). The resulting venous vasculature from a representative dataset is illustrated in Figure 2. In this work, large veins are defined as vessels of sizes similar to that of the superior sagittal sinus, the straight sinus, the inferior sagittal sinus, and the transverse sinus.

#### 2.4.2 Macrovascular signal simulation and correction

The correction approach was detailed in our previous work (X. Z. Zhong, Polimeni, et al., 2024). To briefly recap, as the contribution of the macrovasculature to the BOLD signal is unknown, especially in extravascular voxels, we simulated the macrovascular contribution using biophysical modelling. A diagram summarizing the macrovascular simulation steps is shown in Figure 1b. In our previous work, we discussed the need for a biophysical approach to addressing macrovascular contamination (X. Z. Zhong, Polimeni, et al., 2024). We adapted largely the same simulation process developed in our previous study (methodology development study) (X. Z. Zhong, Polimeni, et al., 2024) to the currently used scan parameters with one minor difference. That is, the original sinusoidal venous input signal was replaced with a more realistic venous input extracted from the current BOLD data, specific to each participant. We identified the prototypical venous signal fluctuation from the in-vivo BOLD signal from the voxel with the maximum fBV, which was then demeaned and normalized by its maximum. As shown in our previous work, this signal can be assumed to be driven primarily by T_2_ fluctuations that mimic the variation in venous blood oxygenation, and can also be assumed to be minimally related to localized neuronal activity (X. Z. Zhong, Polimeni, et al., 2024). The Supplementary Materials section contains additional information about the detailed simulation procedure and mathematical model as we demonstrated in our previous work (X. Z. Zhong, Polimeni, et al., 2024).

Since the simulated macrovascular BOLD signal would not incorporate any time lag among voxels, cross-correlation was applied between each signal and simulated signal at each voxel to determine the lag. This lag associated with the maximum positive cross-correlation coefficient is taken as the time lag between the simulated and in vivo macrovascular signals. It was the lag-corrected simulated signal that was used as the nuisance macrovascular regressor in our voxel-wise linear regression model to reduce the apparent macrovascular BOLD contribution. BOLD signals thus corrected are referred to as the “post-correction” signal.

### 2.5 rs-fMRI preprocessing and analysis

The rs-fMRI data were processed using two different pipelines: one for investigating seed-based functional connectivity (FC) and the other for seed-independent measures. In both pipelines, the first five volumes of BOLD data were rejected to allow the BOLD signal to enter steady state. All metrics were calculated separately for pre- and post-correction BOLD data such that pre- and post-correction inferences were each based on their associated null distributions. Moreover, all metrics below were averaged within each functional network listed in the *Seed-based rs-fMRI analysis* subsection to calculate network-wise metrics.

#### 2.5.1 Seed-based rs-fMRI analysis

rs-fMRI connectivity analysis was performed using the CONN toolbox (Whitfield-Gabrieli & Nieto-Castanon, 2012) with the default_MNI preprocessing pipeline (bandpass filter: 0.01–0.1 Hz; regressed confounds: white matter, CSF, and estimated participant-motion parameters). The networks of interest include the visual network (VN), sensorimotor network (SMN), the default mode network (DMN), the language network (LN), the salience network (SN), the dorsal attention network (DAN), and the frontoparietal network (FPN). The seeds for each network were based on the default seed locations provided in the CONN toolbox. To define each network, given the multiple seeds involved, the correlation maps associated individual seeds, thresholded at p < 0.0001 (FDR corrected), were combined to generate the final network definition.

#### 2.5.2 Seed-independent rs-fMRI analysis

As assessing connectivity based on the seed-based connectivity-defined network boundaries may be somewhat circular, multiple seed-independent approaches have been included. The rs-fMRI preprocessing pipeline was custom designed based on tools from FSL (Jenkinson et al., 2012), AFNI (Cox, 1996), and FreeSurfer (Fischl, 2012). The following steps were included in the preprocessing steps: (a) 3D motion correction (FSL MCFLIRT), (b) slice-timing correction (FSL slicetimer), (c) brain extraction (FSL bet2 and FreeSurfer mri_watershed), (d) rigid body coregistration of functional data to the individual T1 image (FSL FLIRT), (e) regression of the mean signals from white matter (WM) and cerebrospinal fluid (CSF) regions (fsl_glm), (f) bandpass filtering to obtain frequency band 0.01–0.1 Hz (AFNI 3dBandpass), and (g) spatial smoothing with a 6 mm full-width half-maximum (FWHM) Gaussian kernel (FSL fslmaths).

Once the BOLD signal had been preprocessed, we computed the following metrics:

1. Resting-state fluctuation amplitude (RSFA), calculated as the standard deviation of the BOLD signal normalized by the mean of the BOLD signal. This metric reflects the BOLD signal temporal variability that underlies the connectivity measurement.
2. lFCD, calculated using the AFNI (3dLFCD) (Cox, 1996) with a threshold of 0.6, with the neighbourhood data defined to include the six face-touching voxels, as suggested by the previous study (Tomasi & Volkow, 2010). gFCD, calculated using the same threshold value (0.6) but within the entire grey matter (Tomasi & Volkow, 2011). The gFCD and lFCD reflect the number of voxels that each voxel is significantly correlated to (globally or locally). The regions with high gFCD and lFCD have previously been referred to as functional connectivity hubs (Shokri-Kojori et al., 2019; Tomasi & Volkow, 2010, 2011).
3. BOLD signal entropy, calculated as the sample entropy with m = 3 and r = 0.6 related to the standard deviation as proposed by the previous study (Nezafati et al., 2020). The entropy reflects the brain’s capacity for information processing and its adaptability to various cognitive and environmental demands (Wang, 2021) that may not be captured by other linear properties such as gFCD and RSFA.

Since density-related measurements follow an exponential distribution, log-transformed lFCD and gCDF were used in subsequent statistical analyses (Shokri-Kojori et al., 2019).

### 2.6 Baseline perfusion estimation

First, the ASL data were skull stripped, and motion correction was applied to label and control images separately before surround subtraction was performed to determine the signal difference time series. To ensure the brain entered a steady state, the first five volumes of ASL data were also discarded, which is similar to in the rs-fMRI pipeline. The static CBF value was quantified using the BASIL toolbox from FSL (oxford_asl) (Chappell et al., 2009). The static CBF maps were also averaged within each functional network listed in the rs-*fMRI connectivity* subsection to calculate network-based metrics.

Static fractional CBV will be calculated from static CBF by applying linear regression between regionally averaged baseline static CBF (as ml/100 ml/min) and CBV (as a fraction) values in grey and white matter (Eq. 3), measured by previous research with ^15^O steady-state inhalation PET in 34 healthy participants (Leenders et al., 1990; Ma et al., 2020; J. Zhang et al., 2018).

CBV=(0.0723CBF+1.144)/100. (3)

### 2.7 Baseline oxidative metabolism estimation

Both the SE images and the MEGRE image were first skull stripped, and then the rigid-body coregistration was performed to align SE and MEGRE images with the T1-weighted image. Using SE images, R_2_ (refocusable transverse relaxation rate) values were determined based on a single exponential model fitted to each TE. Since the MEGRE scan is sensitive to background fields, a background field correction was performed on the MEGRE data, which calculated the correction factor based on phase components of the MEGRE image and RF pulse shape to estimate gradients in the x, y, and z directions (Kaczmarz et al., 2020). The corrected MEGRE magnitude images were then fitted using a single exponential model to estimate R_2_^*^ (non-refocusable transverse relaxation rate). The R_2_′ was calculated as the difference between R_2_ and R_2_^*^. The OEF and CMRO_2_ were estimated using R_2_′ and perfusion parameters as described in theory. As before, the OEF and CMRO_2_ values were also averaged within each functional network listed in the rs-*fMRI connectivity* subsection to calculate network-wise metrics.

### 2.8 Statistical analysis

We used a linear mixed-effect model (LME) approach to assess the association between network-averaged CMRO_2_ and related physiological metrics and rs-fMRI metrics, both before and after macrovascular correction. In the LME, sex was also considered as a fixed effect of interest since significant sex effects were observed in both static CBF (Mazzucco et al., 2024) and FC metrics (Tomasi & Volkow, 2012). The z-transform was applied to all parameters before fitting. For each fitting, outliers were detected and removed according to the 1.5 interquartile range (IQR) criterion. An overview of the parameters of interest is presented in Table 1 and the model (across all networks and all participants, 140 data points in total) is described as Eq. (4). A significance level of 0.05 was used with a false discovery rate (FDR) correction for results of pre- and post-macrovascular correction separately. The r^2^ values are presented with the number of coefficients adjusted.

**Table 1.** Parameters for linear mixed effect model.

Y ~ 1 + X + Sex + X:Sex (4)	
Y (rs-fMRI)	X (metabolism and hemodynamics)
RSFA	CMRO_2_
Seed-based FC	CBF
gFCD	OEF
lFCD	
Entropy	

In order to better understand the inter-regional variability, a correlation analysis was performed between rs-fMRI metrics and BOLD physiological origins from seven networks for each participant with a significant threshold at p < 0.05. Moreover, to confirm that the associations from the LME model reflect inter-participant differences rather than inter-ROI differences, a similar model with network ID added as a random variable was also assessed. Analysis was also performed on the whole grey matter without separate networks.

## 3 Results

### 3.1 Association between rs-fMRI metrics and physiology

**Fig. 3.**
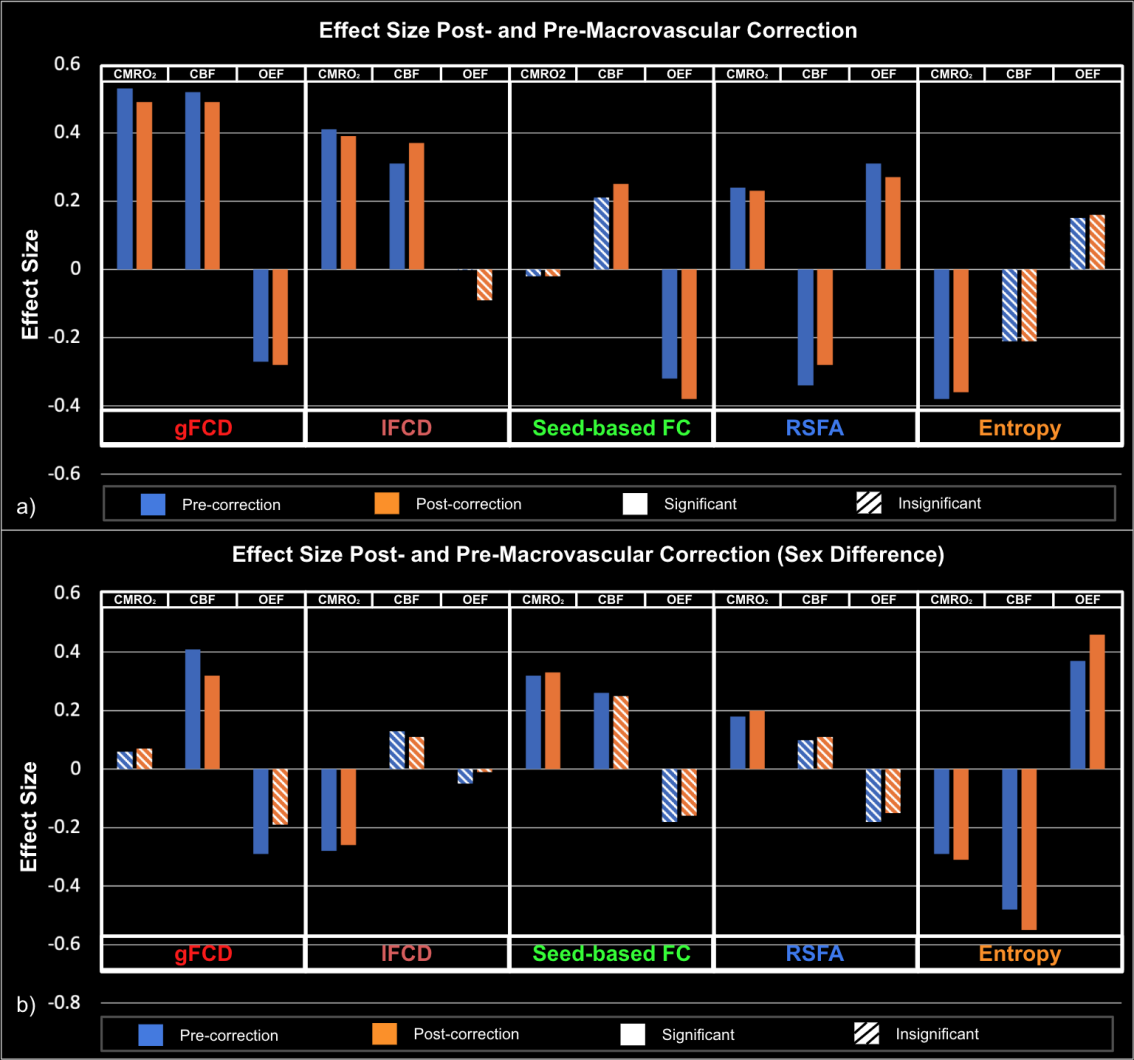
Associations between rs-fMRI metrics and baseline physiological variables (OEF, CBF, and CMRO_2_), with and without accounting for macrovascular correction. (a) Strength of associations between rs-fMRI metrics and baseline physiological variables; (b) comparison of strength of associations between males and females through effect size from the sex interaction effect, with a positive effect size indicating stronger associations in males. Blue: pre-correction; orange: post-correction; striped bars: significant association; dashed bars: insignificant association.

We will first focus on the findings of post-macrovascular correction. The findings in this section are determined across all functional networks. As shown in Figure 3a, all seed-independent rs-fMRI metrics exhibited strong associations with CMRO_2_, however, no seed-based FC was associated with CMRO_2_. Segregated by the direction of the associations, gFCD, lFCD, and RSFA were positively associated with CMRO_2_, while rs-fMRI entropy was negatively associated with CMRO_2_. In comparison with the rest of the rs-fMRI metrics, the gFCD showed a stronger association with CMRO_2_.

OEF and CBF (Fig. 3a) were found to be significantly associated with both seed-based and seed-independent rs-fMRI metrics. OEF was negatively associated with gFCD and seed-based FC and positively associated with RSFA, whereas CBF was positively associated with gFCD, lFCD, seed-based FC, and negatively associated with RSFA. For all baseline physiological variables, we observed negligible differences in effect size with and without the network ID as a random variable. In fact, most of the effect sizes for the network ID random effect were zero. Thus, the associations were indeed dominated by inter-participant differences, and are deemed largely consistent for different networks (Supplementary Table S1).

We found significant sex differences in terms of the associations with CMRO_2_ (Fig. 3b), which suggests the associations between CMRO_2_ and both entropy and lFCD are stronger for females, and the associations between CMRO_2_ and both seed-based FC and RSFA are stronger for males. We also found significant sex differences in terms of the associations with CBF, which suggests the association between CBF and entropy is stronger for females and associations between CBF and gFCD are stronger for males. Moreover, the association between OEF and rs-fMRI entropy is significantly stronger for males. The inter-regional correlations for different participants do not show enough significant results for us to draw conclusions. The next sections consolidate the understanding of the effect of the macrovascular correction.

### 3.2 The effect of macrovascular correction

#### 3.2.1 Comparison of rs-fMRI–physiology associations post- and pre-macrovascular correction

Figure 3 illustrates how the significance of the LMEs linking rs-fMRI and physiological variables was altered due to macrovascular correction. Specifically, (1) the association between seed-based FC and CBF became significant only after macrovascular correction; (2) the sex effect in the association between OEF and gFCD became insignificant after macrovascular correction; (3) the sex effect in the association between seed-based FC and CBF became insignificant after macrovascular correction.

#### 3.2.2 Goodness-of-fit comparison between post- and pre-macrovascular correction

**Fig. 4.**
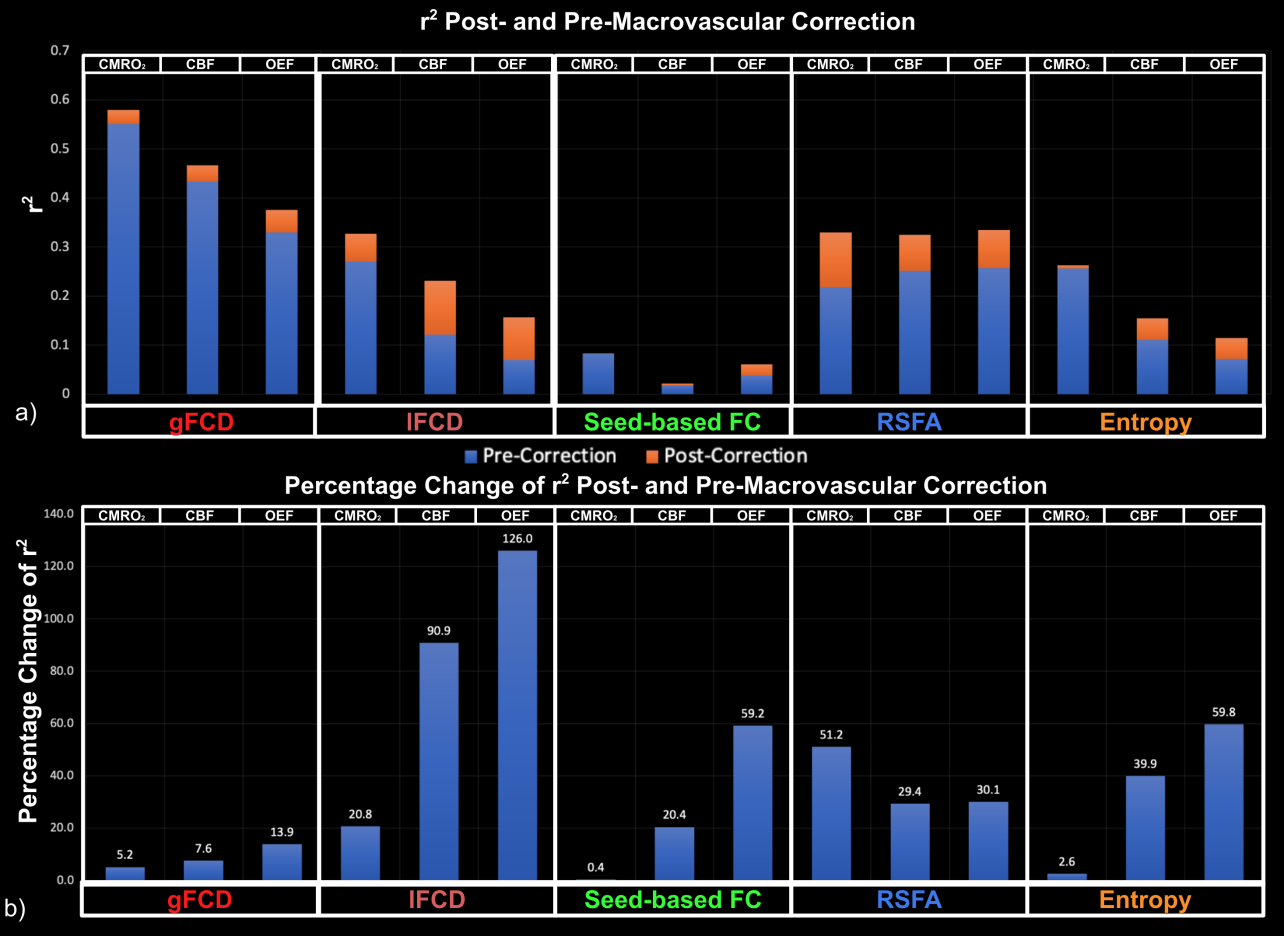
Goodness of fit (r^2^) of LME models, compared between post- and pre-macrovascular correction. (a) r^2^ post- and pre-macrovascular correction; (b) percentage difference in the r^2^ post- and pre-macrovascular correction.

Unlike the effect size shown in the previous subsection, the goodness-of-fit assesses the degree to which the LME fits the data, rather than the contribution of each variable to the model. As shown in Figure 4, in all relationships, the goodness of fit to the LME (as measured by the r^2^) increased when the rs-fMRI metric was based on macrovasculature-corrected data. Notably, the lFCD and RSFA fittings showed the greatest improvement (Fig. 4b), while seed-based FC and entropy fitting (only to CMRO_2_) showed the smallest improvement. Overall, the gFCD–physiology associations were associated with the highest r^2^ of fittings, both pre- and post-correction, followed by the RSFA–physiology associations, with the physiological associations with seed-based FC producing the lowest r^2^ (Fig. 4a). Additionally, as physiological variables go, CMRO_2_ presented a greater r^2^ when fit against rs-fMRI metrics, higher than CBF and OEF.

#### 3.2.3 Comparison of rs-fMRI metrics post- and pre-macrovascular correction

**Fig. 5.**
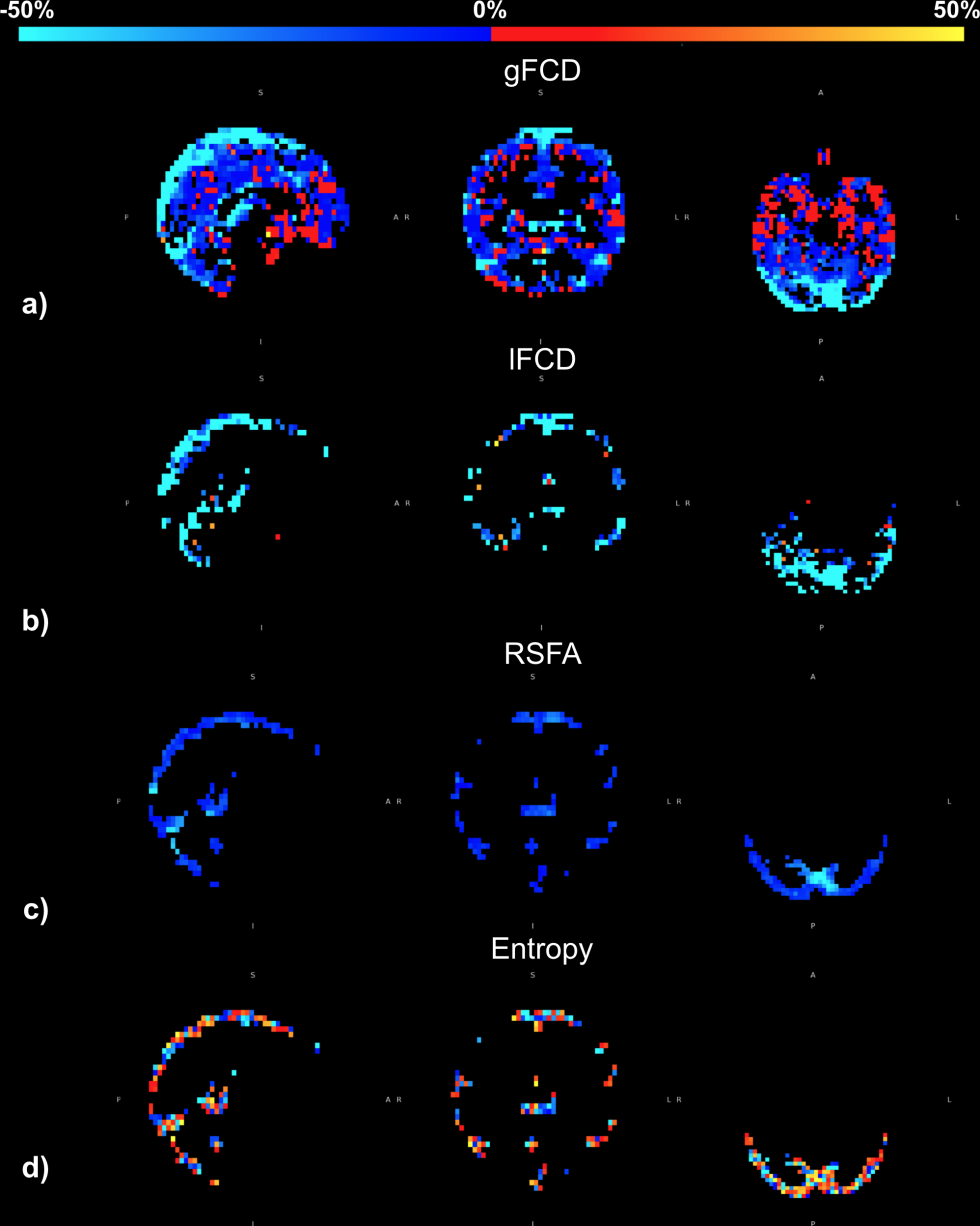
rs-fMRI metrics (network-independent) change after macrovascular correction (data shown from a representative dataset). (a) gFCD; (b) lFCD; (c) RSFA; (d) entropy. The colour bars represent the percentage of change (i.e., (post–pre) as a fraction of the post-correction values).

To better understand the sources of these differences, Figure 5 further quantifies the effect of macrovascular correction on the values of rs-fMRI metrics. The gFCD difference can be seen globally in this representative dataset, with most voxels displaying a decrease in gFCD post-correction (Fig. 5a); the peak of the decreases overlap with the venous vasculature (refer to Fig. 2, especially the superior sagittal sinus). There was also a post-correction decrease in lFCD in most of the voxels (Fig. 5b), but the spatial extent of the decrease was much smaller than for gFCD. A decrease in RSFA occurred after macrovascular correction, primarily in areas surrounding the macrovasculature (Fig. 5c). rs-fMRI entropy varied in both directions post-correction, with a similar spatial extent as that of RSFA, that is, limited to the area surrounding the vasculature (Fig. 5d).

**Fig. 6.**
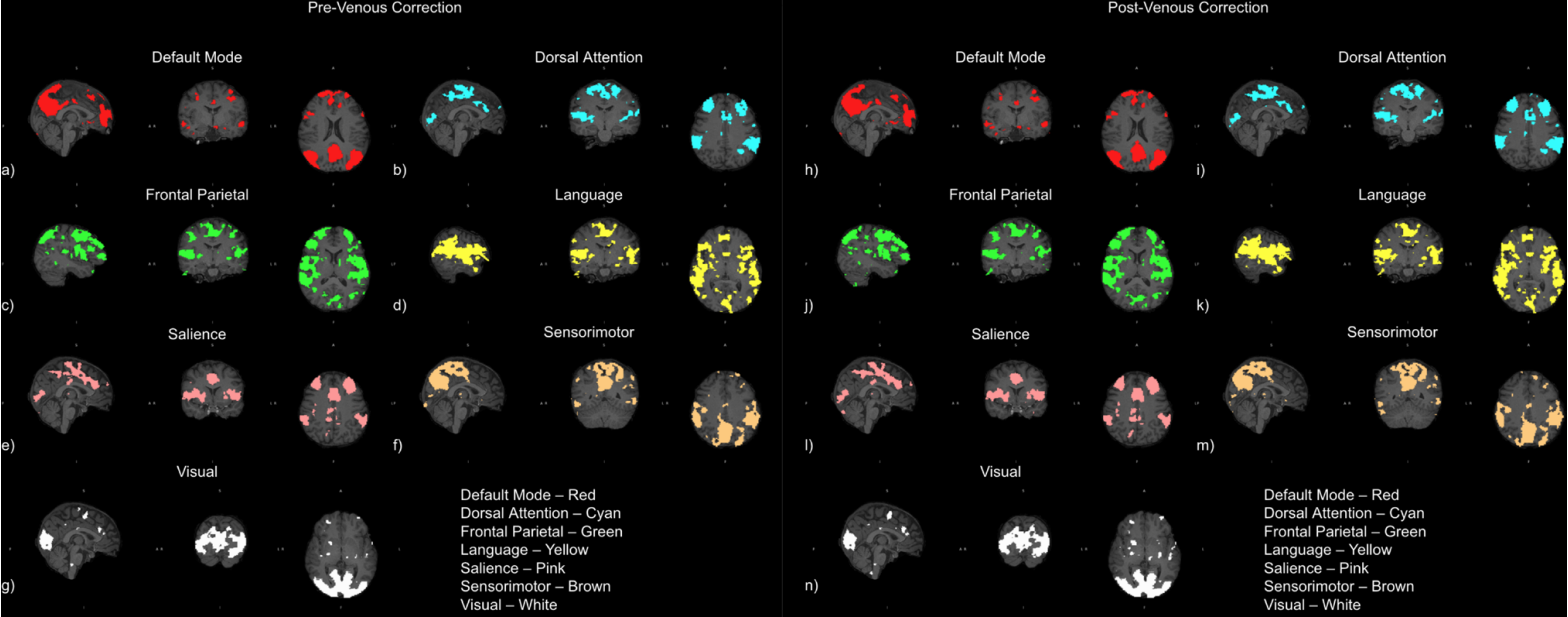
Networks extracted pre- and post-macrovascular correction (Data shown from a single representative dataset). (a-f) pre-correction; (h-m) post-correction.

As described earlier, the network ROI definitions depended on whether macrovascular correction was applied to rs-fMRI data. Thus, to help explain any differences in our findings pre- and post-correction, we also examined differences in the network definitions associated with pre- and post-correction fMRI data. As Figure 6 shows, the spatial extents of the networks extracted pre- and post-macrovascular correction (from a representative participant) showed minimal differences.

## 4 Discussion

The associations between rs-fMRI metrics and fundamental physiological metrics provide a more solid foundation for understanding rs-fMRI findings. Nevertheless, the presence of the venous bias may obscure the associations. As we demonstrated in our previous research, BOLD signal contributions specific to the macrovascular system (particularly the venous system) can be predicted using biophysical modelling (X. Z. Zhong, Polimeni, et al., 2024). In this work, we leveraged this biophysical-modelling approach to minimize the macrovascular contributions, and thereafter we found that

1. The rs-fMRI metrics significantly associated with CMRO_2_ include
  - gFCD, lFCD, and RSFA, which are positively associated with CMRO_2_;
  - rs-fMRI entropy, which is negatively associated with CMRO_2;_
2. The rs-fMRI metrics significantly associated with static CBF include
  - gFCD, lFCD, and seed-based FC, which are positively associated with static CBF;
  - RSFA, which is negatively associated with static CBF;
3. The rs-fMRI metrics significantly associated with OEF include:
  - RSFA, which is positively associated with OEF;
  - gFCD and seed-based FC, which are negatively associated with OEF;
4. The use of macrovascular correction substantially increased the goodness-of-fit of all LME models.

### 4.1 Neuronal activity versus oxidative and glucose metabolism

Previous studies already demonstrated the associations between the rs-fMRI metrics and CMR_glu_ (Shokri-Kojori et al., 2019; Tomasi et al., 2013), but the link between CMRO_glu_ and the BOLD signal is less direct, as similar BOLD responses may be associated with distinct CMR_glu_ responses (Hahn et al., 2024). While oxygen and glucose both serve as fuel for neuronal activity, cerebral glycolytic and oxidative metabolism may not be equally involved. Aerobic metabolism, which produces 38 mol of adenosine triphosphate (ATP) per mol of glucose, is the most efficient way to produce ATP (Krebs & Henseleit, 1932; Morrill, 2021). The entire pathway is composed of two steps: glycolysis in the cytoplasm and Kreb’s cycle in the mitochondria. Glycolysis oxidizes the glucose to pyruvate and produces 2 mol of ATP. In anaerobic conditions, pyruvate is further converted into lactate (Melkonian & Schury, 2024); in aerobic conditions, pyruvate enters the mitochondria and is fully oxidized to carbon dioxide and water through Kerb’s cycle, producing 36 mol ATP. When neuronal activity is in steady state and under normal physiology, it is estimated that 90% of glucose consumption in the brain occurs through oxidative metabolism (molar oxygen–glucose ratio is 5.6) (Gjedde, 2001). However, it is not yet clear whether the coupling between CMR_glu_ and CMRO_2_ would operate in a “steady state” when in resting state.

According to work by Raichle et al. (2001) based on positron emission tomography, the DMN exhibits a higher resting-state CMRO_2_ than the rest of the brain. However, the same cannot be said for CMR_glu_ (Markello et al., 2022). In fact, resting-state CMRO_2_ and CMR_glu_ maps are distinct from one another (Markello et al., 2022). One of the sources of this discrepancy between observed levels of CMR_glu_ and CMRO_2_ metabolism can potentially be explained by aerobic glycolysis (Vaishnavi et al., 2010), which in this context refers to the use of glucose in excess of that required for oxidative phosphorylation in spite of the presence of sufficient oxygen to fully metabolize glucose. Specifically, the area of highest aerobic glycolysis in the resting state was found to overlap with the DMN, which is the most CMRO_2_-demanding region of the brain during the resting state as mentioned earlier, suggesting a mismatch between CMRO_2_ and CMR_glu_. It is evident that CMR_glu_ and static CBF are linked, but a decrease in CBF does not alter CMR_glu_ (Cholet et al., 1997). To summarize, the relationship between rs-fMRI and CMRO_2_, which we focus on in this study, is likely distinct from that with CMRO_glu_.

### 4.2 Associating whole-brain rs-fMRI metrics with static CMRO_2_ and its correlates

The main findings are based on inter-participant differences, as was the case for previous findings relating rs-fMRI to CMR_glu_ (Tomasi et al., 2013). Although this study examined only a limited number of metrics, the conclusions could be extended to other related metrics.

#### 4.2.1 Seed-based FC

We found a significant positive relationship between CBF and seed-based FC, consistent with previous research across participants (Chu et al., 2018; Liang et al., 2013). However, the association between FC and CMRO_2_ was not detected, potentially due to the limited range of FC strength (instead of density) across participants, attributable to the averting of different seed-based FC values (Cole et al., 2010). Moreover, seed-based FC is based on averaged correlation coefficients between seeds within a network, while the seed signal is the averaged signal for a certain brain region (seed region) rather than a voxel-specific signal. Therefore, seed-based approaches may naturally have less local specificity and have a lower association with CMRO_2_, which reflects local-specific neuronal activity.

#### 4.2.2 Seed-independent FC: gFCD and lFCD

As FCD is a measure of how many voxels are connected to a given voxel, and thus the degree of coordination between voxels, regions with a high FCD value are considered connectivity hubs (Tomasi & Volkow, 2010, 2011). It is expected that the maintenance of a higher FCD may require more energy (Raichle & Mintun, 2006). This is indeed what we find, in that higher gFCD and lFCD are associated with higher CMRO_2_. This is consistent with the associations between FCD and CMR_glu_. Tomasi et al. (2013) demonstrated a significant positive correlation between CMRO_glu_ and lFCD and gFCD in the DMN, the DAN across participants, and the cerebellar network and across gray matter (Bernier et al., 2017; Shokri-Kojori et al., 2019). Our study goes a step further by evaluating the link between gFCD, lFCD, and CMRO_2_, while also finding a positive relationship with CBF and a negative one with OEF.

We noticed that the association between CMRO_2_ and FCD (both gFCD and lFCD) is more likely to be driven by CBF, since OEF is negatively associated with FCD. That is, the increased metabolic demands caused by a higher resting-state FCD (both global and local) are overcompensated by an increase in CBF, as is the case in task-related BOLD (Buxton et al., 2004). Though we previously stated that CMRO_2_ and CMR_glu_ might not behave similarly in resting state, they appear to exhibit similar associations with gFCD and lFCD. As such, resting-state cerebral metabolism can be viewed as a form of steady state, as defined previously (in terms of the potential links between CMRO_2_ and CMR_glu_) (Gjedde, 2001).

#### 4.2.3 RSFA

RSFA could reflect both strong neuronal (Leopold et al., 2003; X. Z. Zhong & Chen, 2022) and vascular contributions (Chu et al., 2018; Golestani, Wei, et al., 2016; Tsvetanov et al., 2015), which complicates the interpretation of RSFA. Previous studies have been inconsistent in terms of whether across-participant low-frequency BOLD amplitude is associated with CMR_glu_ (Tomasi et al., 2013) or not (Bernier et al., 2017). We found a significant positive association between RSFA and CMRO_2_ in our study, which mirrors findings linking the RSFA and CMR_glu_ as found by Tomasi et al. (2013). Therefore, RSFA does not merely reflect vascular activity, but also the metabolism associated with local neuronal activity. Compared with connectivity metrics, the strength of associations between CMRO_2_ (as well as OEF and CBF) and RSFA is lower than FCD metrics, but is still stronger than seed-based FC.

Aside from metabolism, baseline CBF is also reportedly significantly associated with the RSFA across participants, the direction of which varies according to the ROI selected (Chu et al., 2018). Results in this study indicate that RSFA is negatively associated with CBF but positively associated with OEF. This could be driven by the nature of RSFA, which is baseline dependent. As illustrated by previous simulations (Chu et al., 2018), a higher baseline CBF as well as a higher resultant blood oxygenation level would both lead to a lower RSFA, irrespective of the underlying neuronal activity. Thus, we do not regard the RSFA as a sufficiently specific metric to assess neuronal activity.

#### 4.2.4 Entropy

Resting-state fMRI entropy is a relatively new metric, which measures the temporal complexity of signals and has been found to negatively correlate with cognitive ability in healthy young adults (Del Mauro & Wang, 2024a), as well as with cortical thickness and surface area in some brain regions (Del Mauro & Wang, 2024b). In previous studies, it was hypothesized that higher entropy represents lower functional temporal coherence and a higher cognitive reserve (Del Mauro & Wang, 2024a, 2024b), which in turn can further transform into a lower metabolic budget (the higher metabolic reserve). This is consistent with our results in that participants with higher rs-fMRI entropy have lower CMRO_2_. The association between rs-fMRI entropy and CMRO_2_ illustrates the neural basis of this temporal coherence metric. In addition, it was reported that correlations between CBF and rs-fMRI entropy are limited (Song et al., 2019), in broad agreement with our findings here. It should be noted, however, that even though the CBF fitting turned out to be insignificant, the effect size is not negligible. Future research that includes more participants may be necessary to fully comprehend these findings.

Entropy showed one of the strongest associations with CMRO_2_, second only to gFCD, albeit in the opposite direction. However, the association of entropy and CBF is poor, suggesting that entropy offers a different perspective on brain function than conventional connectivity metrics.

### 4.3 Sex effects in the rs-fMRI–physiology associations

Sex appears to play a key role in modulating the associations between rs-fMRI metrics and oxidative metabolism. These sex effects are evident across multiple rs-fMRI measures, suggesting that sex may interact differently with distinct aspects of functional connectivity, even when those metrics are conceptually similar.

Physiological differences may underlie these interactions. Females have been shown to exhibit significantly higher CBF than males, whereas sex differences in CMRO_2_ are more modest (Aanerud et al., 2017). Similarly, CMR_glu_ tends to be higher in females, but again the differences are smaller than those observed for CBF (Andreason et al., 1994). These findings suggest that baseline neurovascular and neuro-metabolic coupling may differ by sex: females, though potentially more active metabolically, may extract less oxygen fractionally owing to a larger blood supply. Such differences could alter the relative contribution of OEF and CBF variability to rs-fMRI fluctuations, leading to sex-dependent patterns in the relationship between rs-fMRI and CMRO_2_. Conversely, the metric-specific nature of these interactions may reflect distinct neural or physiological processes captured by different rs-fMRI measures. While the precise mechanisms remain unclear, our previous findings of sex interactions in EEG–fMRI associations (X. Zhong et al., 2025), along with recent reports of sex-modulated CMR_glu_–CBF coupling (Deery et al., 2024), support the existence of systematic sex differences in neurovascular coupling.

Beyond neural activity, non-neuronal factors may also contribute to these sex-dependent associations. For example, microvascular structure has been linked to functional connectivity (Gaudreault & Desjardins, 2025), and if microvascular physiology were to differ by sex, fMRI–CMRO_2_ associations could also differ by sex even in the absence of differences in neurovascular coupling mechanisms. To date, however, direct evidence for sex differences in microvascular anatomy and regulation is lacking. Nonetheless, cerebrovascular reactivity (CVR) differs by sex (Tallon et al., 2020) and has been shown to influence functional connectivity (Chu et al., 2018; Golestani, Kwinta, et al., 2016). It remains unclear to what extent resting-state CVR differences modulate fMRI–CMRO_2_ associations remains an open question.

Collectively, these findings highlight the importance of considering sex as a biological variable in studies of neurovascular coupling. Systematic sex differences in these associations may provide a mechanistic basis for the divergent trajectories of brain aging observed between males and females (Aanerud et al., 2017; Deery et al., 2024; X. Z. Zhong & Chen, 2022). Further investigation is warranted to elucidate the mechanisms underlying these effects.

### 4.4 Macrovascular bias and correction in rs-fMRI

In spite of the fact that both large veins and large arteries have a strong effect on the functional connectivity of the brain (Tong, Yao, et al., 2019; X. Z. Zhong, Tong, et al., 2024), the effect of large veins is much greater than the effect of large arteries (X. Z. Zhong, Tong, et al., 2024). Moreover, predicting the arterial BOLD signal with a biophysical model is more challenging than predicting the venous BOLD signal (X. Z. Zhong, Polimeni, et al., 2024). Since our biophysical model is most reliable for predicting venous and perivenous BOLD signals, this study only addresses venous bias correction.

To begin with, in our previous work (X. Z. Zhong, Polimeni, et al., 2024; X. Z. Zhong, Tong, et al., 2024), we explored in detail how each metric might be affected by macrovascular contribution and how it might change with macrovascular correction. In short, the presence of macrovasculature leads to an overestimation of the RSFA in the regions surrounding the macrovasculature, which in turn is associated with non-linear biases in other rs-fMRI metrics (Chu et al., 2018; X. Z. Zhong, Polimeni, et al., 2024). The resultant connectivity is most likely to be overestimated. The macrovascular influence on fMRI entropy is less amenable to theoretical predictions due to its limited relationship with the RSFA. We used these theoretical predictions to help further establish the validity of the correction through three main arguments.

First, we confirmed the effect of the correction against theoretical predictions. The correction altered the gFCD, lFCD, and RSFA, as illustrated in Figure 5. The RSFA and lFCD, as predicted from theory (Chu et al., 2018; X. Z. Zhong, Polimeni, et al., 2024), decreased. The gFCD also decreases post-correction for the most part. Notably, all reductions closely follow the venous vasculature. However, the gFCD showed a few regional increases as well, which could be the result of a variety of factors. Although the macrovasculature is shown to be strongly correlated within itself (X. Z. Zhong, Polimeni, et al., 2024; X. Z. Zhong, Tong, et al., 2024), it may not always add to the overall FC strength. That is, if the macrovascular signal was to interfere destructively with the neuronal signal, then the overall FC may be reduced due to the macrovascular presence. Another factor contributing to a higher gFCD post-macrovascular correction may be artefactual. As the macrovascular correction is based on a single venous regressor, it is conceivable that venous contributions may be artificially introduced into voxels that have none. Moreover, given that BOLD signals from large arteries tend to be strongly anti-correlated with those from the venous vasculature (X. Z. Zhong, Polimeni, et al., 2024; X. Z. Zhong, Tong, et al., 2024), gFCD can also increase post-correction in the vicinity of arteries. The increase in network-wise entropy after macrovascular correction is intriguing, suggesting that non-linear temporal complexity metrics are not fully immune to physiological contributions, as has been suggested previously (Del Mauro & Wang, 2024a). Further research may be needed to determine whether the current view regarding the interpretation of rs-fMRI entropy stems from the contamination of macrovascular bias.

The second argument is whether macrovascular correction improved the neuronal specificity of rs-fMRI measurements, which reflects the physiological significance for macrovascular correction. It may be possible to answer the question by testing the goodness-of-fit (r^2^) of the association between rs-fMRI metrics and baseline brain physiology metrics, especially CMRO_2_, as explained earlier. A higher r^2^ after macrovascular correction would suggest that rs-fMRI metrics are closer to their BOLD physiological origins and can better reflect local-specific neural activity (Kim et al., 1999; Raichle et al., 2001)—this is exactly what we observed in all cases (Fig. 4). Therefore, these findings support the use of macrovascular correction in the context of BOLD-based rs-fMRI metrics. They also establish the metabolic associations with these multiple rs-fMRI metrics in multiple brain regions, substantially extending previous findings based on CMRO_2_ and CMR_glu_ (Raichle et al., 2001; Shokri-Kojori et al., 2019; Tomasi et al., 2013). It appears that lFCD and RSFA have the most significant improvements in terms of associations with each of the rs-fMRI metrics (Fig. 4). Based on our biophysical model, the RSFA can also be strongly affected by this bias (Ogawa et al., 1993; X. Z. Zhong, Polimeni, et al., 2024). Hence, it is not surprising that associations of RSFA with CMRO_2_ (and its correlates) show strong improvement after macrovascular correction. Moreover, in this regard, the lFCD showed stronger improvements than gFCD, as the definition of the lFCD neighbourhoods favours neighbouring voxels with higher correlation, which fit the behaviour of voxels containing large vessels. In contrast, seed-based FC shows very limited improvement following macrovascular correction (Fig. 4), but as seed-based FC shows an inherently low association with CMRO_2_ and low r^2^ (see Seed-based FC section), it is deemed not the best surrogate for neuronal metabolism based on our findings. The extent to which venous bias affects the measurement of entropy remains unclear, but it is also the case that we do not understand the entropic behaviour of macrovascular BOLD.

The final argument for macrovascular correction is the value added to rs-fMRI analyses as a result of the correction. We provide evidence that certain relationships can only be observed after the correction. For example, the relationship between seed-based FC and CBF is only significant after macrovascular correction has been applied (Fig. 3). It is also important to note that with more participants, the difference between pre- and post-macrovascular correction could be even more evident. Additionally, some associations were eliminated after the macrovascular correction. Some examples include the sex dependence of the gFCD-OEF association and of the seed-based FC-CBF association (Fig. 3). The correction did not strengthen any sex dependence in the rs-fMRI–physiology associations. As we cannot justify the association between rs-fMRI and physiology being sex dependent, these findings may be a sign that some of the associations that we previously found may have been caused by venous bias rather than neuronal activity.

### 4.5 Recommendation

Our current study goes beyond our previous studies (X. Z. Zhong, Polimeni, et al., 2024) by applying the biophysical modelling-based macrovascular correction and validating its effectiveness. We would like to reiterate the recommendations that we made in our previous article that TOF images should be collected along with the BOLD measurements (X. Z. Zhong, Tong, et al., 2024) and that a macro-VAN model can be used to predict venous behaviour with great accuracy (X. Z. Zhong, Tong, et al., 2024). Moreover, we show that it is feasible to use the BOLD signal from the voxel with maximum fBV as a surrogate for Yv variation in the simulation of the biophysical model. Furthermore, venous signal contributions could be corrected through regression with a lag shift in the simulated venous signal, as we outlined here. More sophisticated correction approaches (for example, principal component analysis (PCA) and independent component analysis (ICA)) will be examined in future research. It is also important to note that null statistical distributions may differ pre- and post-correction, which should be taken into consideration when performing the analysis. Lastly, based on these findings, we recommend that macrovascular corrections be applied to all rs-fMRI experiments focusing on localized neuronal interpretations. More broadly, our methods can be applied to all fMRI studies. However, greater caution needs to be exercised for experiments focusing on more global BOLD effects (such as sleep studies and autonomic nervous system studies), as the interpretation of the macrovascular signals may be less clear in such cases.

### 4.6 Limitations

Limitations directly related to TOF imaging acquisition and marco-VAN model simulation have already been addressed in our previous paper (X. Z. Zhong, Polimeni, et al., 2024; X. Z. Zhong, Tong, et al., 2024) and will not be repeated here. We want to emphasize that we did not include arterial bias in the correction. Further, the CBV used in this study is based on a linear relationship with whole-brain CBF, but CBF may vary across different networks. Unfortunately, we were unable to evaluate this association in each network based on our data. Moreover, this study focuses on the feasibility and validation of our macrovascular correction approach, and thus does not include a comprehensive comparison with previous macrovascular correction approaches. As well, a relatively shorter scan has been used in order to minimize computational demands for macrovascular simulation, which could potentially lead to errors in connectivity estimation. In spite of this, the spatial patterns of our lFCD and gFCD are comparable with those reported previously by Tomasi and Volkow (2010, 2011), lending confidence of the generalizability of our findings. While we advocate for macrovascular correction on the merits of a stronger resultant association with CMRO_2_, macrovascular contributions to CMRO_2_ cannot be completely eliminated. MRI-based CMRO_2_ measurements are also sensitive to macrovascular contributions, considering they are also based on R_2_′ contrast. Although the OEF estimates are based on bias-field corrected R_2_′ estimates, the effect of venous bias could remain. Thus, while using CMRO_2_ and OEF to test the effect of rs-fMRI macrovascular correction is a major step forward in establishing the neuronal implications of rs-fMRI, non-MRI-based validation (for example, using EEG) would also need to be considered.

Another potential concern is the use of atypical fMRI scan parameters in this study, including a relatively short scan time and a higher flip angle, which may increase the inflow effect of macrovascular bias. Recent findings have further suggested that high-frequency fMRI signals can be significantly associated with neuroelectrophysiological measurements (Lewis et al., 2016) and potentially metabolic measurements. However, we currently do not have sufficient evidence to determine whether changes in scan parameters would strengthen or weaken the associations reported here. Examining the generalizability of our results across different scanning settings would be an interesting topic for future work.

## 5 Conclusion

Clinical applications of rs-fMRI are challenging as there is no clear understanding of the physiological mechanisms that underlie rs-fMRI metrics. In order to make the rs-fMRI metrics more interpretable, it would be necessary to understand the relationship between rs-fMRI metrics and CMRO_2_, which is directly related to neuronal activity. This study helps establish these associations across multiple brain regions, as well as demonstrates how our novel macrovascular correction can enhance these associations. As we show the interpretability of the rs-fMRI metrics being further improved following macrovascular correction, we suggest that macrovascular correction enhances local-neuronal specificity of rs-fMRI metrics. Results presented in this article may be used to improve the interpretability of rs-fMRI metrics in the future, as well as to promote its clinical applications.

## Supplementary Material

Supplementary Material

## Data Availability

The data and code will be made available upon request.

## References

[IMAG.a.1060-b1] Aanerud, J., Borghammer, P., Rodell, A., Jónsdottir, K. Y., & Gjedde, A. (2017). Sex differences of human cortical blood flow and energy metabolism. Journal of Cerebral Blood Flow and Metabolism: Official Journal of the International Society of Cerebral Blood Flow and Metabolism, 37(7), 2433–2440. 10.1177/0271678X16668536 27629099 PMC5531342

[IMAG.a.1060-b2] An, H., Lin, W., Celik, A., & Lee, Y. Z. (2001). Quantitative measurements of cerebral metabolic rate of oxygen utilization using MRI: A volunteer study. NMR in Biomedicine, 14(7–8), 441–447. 10.1002/nbm.717 11746936 PMC4096838

[IMAG.a.1060-b3] Andreason, P. J., Zametkin, A. J., Guo, A. C., Baldwin, P., & Cohen, R. M. (1994). Gender-related differences in regional cerebral glucose metabolism in normal volunteers. Psychiatry Research, 51(2), 175–183. 10.1016/0165-1781(94)90037-x 8022952

[IMAG.a.1060-b4] Bernier, M., Croteau, E., Castellano, C.-A., Cunnane, S. C., & Whittingstall, K. (2017). Spatial distribution of resting-state BOLD regional homogeneity as a predictor of brain glucose uptake: A study in healthy aging. NeuroImage, 150, 14–22. 10.1016/j.neuroimage.2017.01.055 28130193

[IMAG.a.1060-b5] Bernier, M., Cunnane, S. C., & Whittingstall, K. (2018). The morphology of the human cerebrovascular system. Human Brain Mapping, 39(12), 4962–4975. 10.1002/hbm.24337 30265762 PMC6866388

[IMAG.a.1060-b6] Biswal, B., Yetkin, F. Z., Haughton, V. M., & Hyde, J. S. (1995). Functional connectivity in the motor cortex of resting human brain using echo-planar MRI. Magnetic Resonance in Medicine, 34(4), 537–541. 10.1002/mrm.1910340409 8524021

[IMAG.a.1060-b7] Biswal, B. B., Van Kylen, J., & Hyde, J. S. (1997). Simultaneous assessment of flow and BOLD signals in resting-state functional connectivity maps. NMR in Biomedicine, 10(4–5), 165–170. 10.1002/(sici)1099-1492(199706/08)10:4/5<165::aid-nbm454>3.0.co;2-7 9430343

[IMAG.a.1060-b8] Boxerman, J. L., Hamberg, L. M., Rosen, B. R., & Weisskoff, R. M. (1995). MR contrast due to intravascular magnetic susceptibility perturbations. Magnetic Resonance in Medicine, 34(4), 555–566. 10.1002/mrm.1910340412 8524024

[IMAG.a.1060-b9] Buxton, R. B., Uludağ, K., Dubowitz, D. J., & Liu, T. T. (2004). Modeling the hemodynamic response to brain activation. NeuroImage, 23(Suppl. 1), S220–S233. 10.1016/j.neuroimage.2004.07.013 15501093

[IMAG.a.1060-b10] Chappell, M. A., Groves, A. R., Whitcher, B., & Woolrich, M. W. (2009). Variational Bayesian inference for a nonlinear forward model. IEEE Transactions on Signal Processing: A Publication of the IEEE Signal Processing Society, 57(1), 223–236. 10.1109/tsp.2008.2005752

[IMAG.a.1060-b11] Cholet, N., Seylaz, J., Lacombe, P., & Bonvento, G. (1997). Local uncoupling of the cerebrovascular and metabolic responses to somatosensory stimulation after neuronal nitric oxide synthase inhibition. Journal of Cerebral Blood Flow and Metabolism: Official Journal of the International Society of Cerebral Blood Flow and Metabolism, 17(11), 1191–1201. 10.1097/00004647-199711000-00008 9390651

[IMAG.a.1060-b12] Chu, P. P. W., Golestani, A. M., Kwinta, J. B., Khatamian, Y. B., & Chen, J. J. (2018). Characterizing the modulation of resting-state fMRI metrics by baseline physiology. NeuroImage, 173, 72–87. 10.1016/j.neuroimage.2018.02.004 29452265

[IMAG.a.1060-b13] Cole, M. W., Pathak, S., & Schneider, W. (2010). Identifying the brain’s most globally connected regions. NeuroImage, 49(4), 3132–3148. 10.1016/j.neuroimage.2009.11.001 19909818

[IMAG.a.1060-b14] Cox, R. W. (1996). AFNI: Software for analysis and visualization of functional magnetic resonance neuroimages. Computers and Biomedical Research, an International Journal, 29(3), 162–173. 10.1006/cbmr.1996.0014 8812068

[IMAG.a.1060-b15] Davis, T. L., Kwong, K. K., Weisskoff, R. M., & Rosen, B. R. (1998). Calibrated functional MRI: Mapping the dynamics of oxidative metabolism. Proceedings of the National Academy of Sciences of the United States of America, 95(4), 1834–1839. 10.1073/pnas.95.4.1834 9465103 PMC19199

[IMAG.a.1060-b16] Deery, H., Moran, C., Liang, E. X., Gurvich, C., Egan, G. F., & Jamadar, S. D. (2024). Sex differences in the rates and association of cerebral blood flow and glucose metabolism in normative ageing. bioRxiv. https://www.biorxiv.org/content/10.1101/2024.11.27.625794v2.full.pdf 10.1002/hbm.70328 PMC12371555 40844193

[IMAG.a.1060-b17] Del Mauro, G., & Wang, Z. (2024a). Associations of brain entropy estimated by resting state fMRI with physiological indices, body mass index, and cognition. Journal of Magnetic Resonance Imaging: JMRI, 59(5), 1697–1707. 10.1002/jmri.28948 37578314 PMC10864678

[IMAG.a.1060-b18] Del Mauro, G., & Wang, Z. (2024b). rsfMRI-based brain entropy is negatively correlated with gray matter volume and surface area. bioRxiv. 10.1101/2024.04.28.59137139869211

[IMAG.a.1060-b19] Fischl, B. (2012). FreeSurfer. NeuroImage, 62(2), 774–781. 10.1016/j.neuroimage.2012.01.021 22248573 PMC3685476

[IMAG.a.1060-b20] Gal, S., Coldham, Y., Tik, N., Bernstein-Eliav, M., & Tavor, I. (2022). Act natural: Functional connectivity from naturalistic stimuli fMRI outperforms resting-state in predicting brain activity. NeuroImage, 258, 119359. 10.1016/j.neuroimage.2022.119359 35680054

[IMAG.a.1060-b21] Gaudreault, F., & Desjardins, M. (2025). Microvascular structure variability explains variance in fMRI functional connectivity. Brain Structure & Function, 230(2), 39. 10.1007/s00429-025-02899-4 39921726

[IMAG.a.1060-b22] Gibbs, E. L., Lennox, W. G., Nims, L. F., & Gibbs, F. A. (1942). Arterial and cerebral venous blood. The Journal of Biological Chemistry, 144(2), 325–332. 10.1016/s0021-9258(18)72512-x

[IMAG.a.1060-b23] Gjedde, A. (2001). Brain energy metabolism and the physiological basis of the haemodynamic response. In P. Jezzard, P. M. Matthews, & S. M. Smith (Eds.), Functional magnetic resonance imaging (pp. 38–67). Oxford University Press. 10.1093/acprof:oso/9780192630711.003.0002

[IMAG.a.1060-b24] Golestani, A. M., Kwinta, J. B., Strother, S. C., Khatamian, Y. B., & Chen, J. J. (2016). The association between cerebrovascular reactivity and resting-state fMRI functional connectivity in healthy adults: The influence of basal carbon dioxide. NeuroImage, 132, 301–313. 10.1016/j.neuroimage.2016.02.051 26908321 PMC5148617

[IMAG.a.1060-b25] Golestani, A. M., Wei, L. L., & Chen, J. J. (2016). Quantitative mapping of cerebrovascular reactivity using resting-state BOLD fMRI: Validation in healthy adults. NeuroImage, 138, 147–163. 10.1016/j.neuroimage.2016.05.025 27177763 PMC5148619

[IMAG.a.1060-b26] Göttler, J., Kaczmarz, S., Kallmayer, M., Wustrow, I., Eckstein, H.-H., Zimmer, C., Sorg, C., Preibisch, C., & Hyder, F. (2019). Flow-metabolism uncoupling in patients with asymptomatic unilateral carotid artery stenosis assessed by multi-modal magnetic resonance imaging. Journal of Cerebral Blood Flow and Metabolism: Official Journal of the International Society of Cerebral Blood Flow and Metabolism, 39(11), 2132–2143. 10.1177/0271678X18783369 29968499 PMC6827123

[IMAG.a.1060-b27] Hahn, A., Reed, M. B., Vraka, C., Godbersen, G. M., Klug, S., Komorowski, A., Falb, P., Nics, L., Traub-Weidinger, T., Hacker, M., & Lanzenberger, R. (2024). High-temporal resolution functional PET/MRI reveals coupling between human metabolic and hemodynamic brain response. European Journal of Nuclear Medicine and Molecular Imaging, 51(5), 1310–1322. 10.1007/s00259-023-06542-4 38052927 PMC11399190

[IMAG.a.1060-b28] Hirsch, N. M., Toth, V., Förschler, A., Kooijman, H., Zimmer, C., & Preibisch, C. (2014). Technical considerations on the validity of blood oxygenation level-dependent-based MR assessment of vascular deoxygenation. NMR in Biomedicine, 27(7), 853–862. 10.1002/nbm.3131 24809665

[IMAG.a.1060-b29] Hoge, R. D., Atkinson, J., Gill, B., Crelier, G. R., Marrett, S., & Pike, G. B. (1999). Investigation of BOLD signal dependence on cerebral blood flow and oxygen consumption: The deoxyhemoglobin dilution model. Magnetic Resonance in Medicine, 42(5), 849–863. 10.1002/(sici)1522-2594(199911)42:5<849::aid-mrm4>3.0.co;2-z 10542343

[IMAG.a.1060-b30] Hyde, J. S., & Li, R. (2014). Functional connectivity in rat brain at 200 μm resolution. Brain Connectivity, 4(7), 470–480. 10.1089/brain.2014.0281 25112943 PMC4146383

[IMAG.a.1060-b31] Jenkinson, M., Beckmann, C. F., Behrens, T. E. J., Woolrich, M. W., & Smith, S. M. (2012). FSL. NeuroImage, 62(2), 782–790. 10.1016/j.neuroimage.2011.09.015 21979382

[IMAG.a.1060-b32] Kaczmarz, S., Hyder, F., & Preibisch, C. (2020). Oxygen extraction fraction mapping with multi-parametric quantitative BOLD MRI: Reduced transverse relaxation bias using 3D-GraSE imaging. NeuroImage, 220, 117095. 10.1016/j.neuroimage.2020.117095 32599265 PMC7730517

[IMAG.a.1060-b33] Kannurpatti, S. S., Motes, M. A., Rypma, B., & Biswal, B. B. (2011). Increasing measurement accuracy of age-related BOLD signal change: Minimizing vascular contributions by resting-state-fluctuation-of-amplitude scaling. Human Brain Mapping, 32(7), 1125–1140. 10.1002/hbm.21097 20665721 PMC3310892

[IMAG.a.1060-b34] Kim, S. G., Rostrup, E., Larsson, H. B., Ogawa, S., & Paulson, O. B. (1999). Determination of relative CMRO2 from CBF and BOLD changes: Significant increase of oxygen consumption rate during visual stimulation. Magnetic Resonance in Medicine, 41(6), 1152–1161. 10.1002/(sici)1522-2594(199906)41:6<1152::aid-mrm11>3.0.co;2-t 10371447

[IMAG.a.1060-b35] Krebs, H. A., & Henseleit, K. (1932). Untersuchungen uber die Harnstoffbildung im Tierkörper. Hoppe-Seyler’s Zeitschrift Fur Physiologische Chemie, 210(1–2), 33–66. 10.1515/bchm2.1932.210.1-2.33

[IMAG.a.1060-b36] Lee, M. H., Smyser, C. D., & Shimony, J. S. (2013). Resting-state fMRI: A review of methods and clinical applications. AJNR. American Journal of Neuroradiology, 34(10), 1866–1872. 10.3174/ajnr.A3263 22936095 PMC4035703

[IMAG.a.1060-b37] Leenders, K. L., Perani, D., Lammertsma, A. A., Heather, J. D., Buckingham, P., Healy, M. J., Gibbs, J. M., Wise, R. J., Hatazawa, J., & Herold, S. (1990). Cerebral blood flow, blood volume and oxygen utilization. Normal values and effect of age. Brain: A Journal of Neurology, 113(Pt 1), 27–47. 10.1093/brain/113.1.27 2302536

[IMAG.a.1060-b38] Leopold, D. A., Murayama, Y., & Logothetis, N. K. (2003). Very slow activity fluctuations in monkey visual cortex: Implications for functional brain imaging. Cerebral Cortex, 13(4), 422–433. 10.1093/cercor/13.4.422 12631571

[IMAG.a.1060-b39] Lewis, L. D., Setsompop, K., Rosen, B. R., & Polimeni, J. R. (2016). Fast fMRI can detect oscillatory neural activity in humans. Proceedings of the National Academy of Sciences of the United States of America, 113(43), E6679–E6685. 10.1073/pnas.1608117113 27729529 PMC5087037

[IMAG.a.1060-b40] Liang, X., Zou, Q., He, Y., & Yang, Y. (2013). Coupling of functional connectivity and regional cerebral blood flow reveals a physiological basis for network hubs of the human brain. Proceedings of the National Academy of Sciences of the United States of America, 110(5), 1929–1934. 10.1073/pnas.1214900110 23319644 PMC3562840

[IMAG.a.1060-b41] Ma, Y., Sun, H., Cho, J., Mazerolle, E. L., Wang, Y., & Pike, G. B. (2020). Cerebral OEF quantification: A comparison study between quantitative susceptibility mapping and dual-gas calibrated BOLD imaging. Magnetic Resonance in Medicine, 83(1), 68–82. 10.1002/mrm.27907 31373088

[IMAG.a.1060-b42] Markello, R. D., Hansen, J. Y., Liu, Z.-Q., Bazinet, V., Shafiei, G., Suárez, L. E., Blostein, N., Seidlitz, J., Baillet, S., Satterthwaite, T. D., Chakravarty, M. M., Raznahan, A., & Misic, B. (2022). Neuromaps: Structural and functional interpretation of brain maps. Nature Methods, 19(11), 1472–1479. 10.1038/s41592-022-01625-w 36203018 PMC9636018

[IMAG.a.1060-b43] Mazzucco, S., Li, L., Tuna, M. A., & Rothwell, P. M. (2024). Age-specific sex-differences in cerebral blood flow velocity in relation to haemoglobin levels. European Stroke Journal, 9(3), 772–780. 10.1177/23969873241245631 38634499 PMC11343687

[IMAG.a.1060-b44] Melkonian, E. A., & Schury, M. P. (2024). Biochemistry, anaerobic glycolysis. In StatPearls. StatPearls Publishing. https://www.ncbi.nlm.nih.gov/books/NBK546695/31536301

[IMAG.a.1060-b45] Menon, R. S. (2012). The great brain versus vein debate. NeuroImage, 62(2), 970–974. 10.1016/j.neuroimage.2011.09.005 21939776

[IMAG.a.1060-b46] Menon, R. S., & Goodyear, B. G. (1999). Submillimeter functional localization in human striate cortex using BOLD contrast at 4 Tesla: Implications for the vascular point-spread function. Magnetic Resonance in Medicine: Official Journal of the Society of Magnetic Resonance in Medicine / Society of Magnetic Resonance in Medicine, 41(2), 230–235. 10.1002/(sici)1522-2594(199902)41:2<230::aid-mrm3>3.0.co;2-o 10080267

[IMAG.a.1060-b47] Morrill, J. S. (2021, December 14). 11.6: Aerobic Metabolism and the Mitochondria. Medicine LibreTexts; Libretexts. https://med.libretexts.org/Bookshelves/Nutrition/Realities_of_Nutrition_%28Morrill%29/06%3A_Fueling_the_Body/11%3A_Metabolism_and_the_Vitamin_Key/11.06%3A_Aerobic_Metabolism_and_the_Mitochondria

[IMAG.a.1060-b48] Nezafati, M., Temmar, H., & Keilholz, S. D. (2020). Functional MRI signal complexity analysis using sample entropy. Frontiers in Neuroscience, 14, 700. 10.3389/fnins.2020.00700 32714141 PMC7344022

[IMAG.a.1060-b49] Ogawa, S., Menon, R. S., Tank, D. W., Kim, S. G., Merkle, H., Ellermann, J. M., & Ugurbil, K. (1993). Functional brain mapping by blood oxygenation level-dependent contrast magnetic resonance imaging. A comparison of signal characteristics with a biophysical model. Biophysical Journal, 64(3), 803–812. 10.1016/s0006-3495(93)81441-3 8386018 PMC1262394

[IMAG.a.1060-b50] Phadikar, S., Pusuluri, K., Iraji, A., & Calhoun, V. D. (2025). Integrating fMRI spatial network dynamics and EEG spectral power: Insights into resting state connectivity. Frontiers in Neuroscience, 19, 1484954. 10.3389/fnins.2025.1484954 39935841 PMC11810936

[IMAG.a.1060-b51] Raichle, M. E., MacLeod, A. M., Snyder, A. Z., Powers, W. J., Gusnard, D. A., & Shulman, G. L. (2001). A default mode of brain function. Proceedings of the National Academy of Sciences of the United States of America, 98(2), 676–682. 10.1073/pnas.98.2.676 11209064 PMC14647

[IMAG.a.1060-b52] Raichle, M. E., & Mintun, M. A. (2006). Brain work and brain imaging. Annual Review of Neuroscience, 29, 449–476. 10.1146/annurev.neuro.29.051605.112819 16776593

[IMAG.a.1060-b53] Scherr, M., Utz, L., Tahmasian, M., Pasquini, L., Grothe, M. J., Rauschecker, J. P., Grimmer, T., Drzezga, A., Sorg, C., & Riedl, V. (2021). Effective connectivity in the default mode network is distinctively disrupted in Alzheimer’s disease-A simultaneous resting-state FDG-PET/fMRI study. Human Brain Mapping, 42(13), 4134–4143. 10.1002/hbm.24517 30697878 PMC8357005

[IMAG.a.1060-b54] Schimmelpfennig, J., Topczewski, J., Zajkowski, W., & Jankowiak-Siuda, K. (2023). The role of the salience network in cognitive and affective deficits. Frontiers in Human Neuroscience, 17, 1133367. 10.3389/fnhum.2023.1133367 37020493 PMC10067884

[IMAG.a.1060-b55] Shokri-Kojori, E., Tomasi, D., Alipanahi, B., Wiers, C. E., Wang, G.-J., & Volkow, N. D. (2019). Correspondence between cerebral glucose metabolism and BOLD reveals relative power and cost in human brain. Nature Communications, 10(1), 690. 10.1038/s41467-019-08546-x PMC6370887 30741935

[IMAG.a.1060-b56] Song, D., Chang, D., Zhang, J., Ge, Q., Zang, Y.-F., & Wang, Z. (2019). Associations of brain entropy (BEN) to cerebral blood flow and fractional amplitude of low-frequency fluctuations in the resting brain. Brain Imaging and Behavior, 13(5), 1486–1495. 10.1007/s11682-018-9963-4 30209786

[IMAG.a.1060-b57] Tallon, C. M., Barker, A. R., Nowak-Flück, D., Ainslie, P. N., & McManus, A. M. (2020). The influence of age and sex on cerebrovascular reactivity and ventilatory response to hypercapnia in children and adults. Experimental Physiology, 105(7), 1090–1101. 10.1113/EP088293 32333697

[IMAG.a.1060-b58] Tomasi, D., & Volkow, N. D. (2010). Functional connectivity density mapping. Proceedings of the National Academy of Sciences of the United States of America, 107(21), 9885–9890. 10.1073/pnas.1001414107 20457896 PMC2906909

[IMAG.a.1060-b59] Tomasi, D., & Volkow, N. D. (2011). Functional connectivity hubs in the human brain. NeuroImage, 57(3), 908–917. 10.1016/j.neuroimage.2011.05.024 21609769 PMC3129362

[IMAG.a.1060-b60] Tomasi, D., & Volkow, N. D. (2012). Laterality patterns of brain functional connectivity: Gender effects. Cerebral Cortex, 22(6), 1455–1462. 10.1093/cercor/bhr230 21878483 PMC3450858

[IMAG.a.1060-b61] Tomasi, D., Wang, G.-J., & Volkow, N. D. (2013). Energetic cost of brain functional connectivity. Proceedings of the National Academy of Sciences of the United States of America, 110(33), 13642–13647. 10.1073/pnas.1303346110 23898179 PMC3746878

[IMAG.a.1060-b62] Tong, Y., Hocke, L. M., Fan, X., Janes, A. C., & Frederick, B. D. (2015). Can apparent resting state connectivity arise from systemic fluctuations? Frontiers in Human Neuroscience, 9, 285. 10.3389/fnhum.2015.00285 26029095 PMC4432665

[IMAG.a.1060-b63] Tong, Y., Hocke, L. M., & Frederick, B. B. (2019). Low frequency systemic hemodynamic “noise” in resting state BOLD fMRI: Characteristics, causes, implications, mitigation strategies, and applications. Frontiers in Neuroscience, 13, 787. 10.3389/fnins.2019.00787 31474815 PMC6702789

[IMAG.a.1060-b64] Tong, Y., Yao, J. F., Chen, J. J., & Frederick, B. D. (2019). The resting-state fMRI arterial signal predicts differential blood transit time through the brain. Journal of Cerebral Blood Flow and Metabolism: Official Journal of the International Society of Cerebral Blood Flow and Metabolism, 39(6), 1148–1160. 10.1177/0271678X17753329 29333912 PMC6547182

[IMAG.a.1060-b65] Tortora, G. J., & Derrickson, B. H. (2018). Principles of anatomy and physiology. John Wiley & Sons. https://play.google.com/store/books/details?id=aSaVDwAAQBAJ

[IMAG.a.1060-b66] Tsvetanov, K. A., Henson, R. N. A., Tyler, L. K., Davis, S. W., Shafto, M. A., Taylor, J. R., Williams, N., Cam-Can, & Rowe, J. B. (2015). The effect of ageing on fMRI: Correction for the confounding effects of vascular reactivity evaluated by joint fMRI and MEG in 335 adults. Human Brain Mapping, 36(6), 2248–2269. 10.1002/hbm.22768 25727740 PMC4730557

[IMAG.a.1060-b67] Vaishnavi, S. N., Vlassenko, A. G., Rundle, M. M., Snyder, A. Z., Mintun, M. A., & Raichle, M. E. (2010). Regional aerobic glycolysis in the human brain. Proceedings of the National Academy of Sciences of the United States of America, 107(41), 17757–17762. 10.1073/pnas.1010459107 20837536 PMC2955101

[IMAG.a.1060-b68] Wang, Z. (2021). The neurocognitive correlates of brain entropy estimated by resting state fMRI. NeuroImage, 232, 117893. 10.1016/j.neuroimage.2021.117893 33621695 PMC8138544

[IMAG.a.1060-b69] Whitfield-Gabrieli, S., & Nieto-Castanon, A. (2012). Conn: A functional connectivity toolbox for correlated and anticorrelated brain networks. Brain Connectivity, 2(3), 125–141. 10.1089/brain.2012.0073 22642651

[IMAG.a.1060-b70] Wu, C. W., Gu, H., Lu, H., Stein, E. A., Chen, J.-H., & Yang, Y. (2009). Mapping functional connectivity based on synchronized CMRO2 fluctuations during the resting state. NeuroImage, 45(3), 694–701. 10.1016/j.neuroimage.2008.12.066 19280693 PMC2775537

[IMAG.a.1060-b71] Yablonskiy, D. A., & Haacke, E. M. (1994). Theory of NMR signal behavior in magnetically inhomogeneous tissues: The static dephasing regime. Magnetic Resonance in Medicine, 32(6), 749–763. 10.1002/mrm.1910320610 7869897

[IMAG.a.1060-b72] Yu, X., Glen, D., Wang, S., Dodd, S., Hirano, Y., Saad, Z., Reynolds, R., Silva, A. C., & Koretsky, A. P. (2012). Direct imaging of macrovascular and microvascular contributions to BOLD fMRI in layers IV–V of the rat whisker–barrel cortex. NeuroImage, 59(2), 1451–1460. 10.1016/j.neuroimage.2011.08.001 21851857 PMC3230765

[IMAG.a.1060-b73] Zhang, J., Cho, J., Zhou, D., Nguyen, T. D., Spincemaille, P., Gupta, A., & Wang, Y. (2018). Quantitative susceptibility mapping-based cerebral metabolic rate of oxygen mapping with minimum local variance. Magnetic Resonance in Medicine, 79(1), 172–179. 10.1002/mrm.26657 28295523

[IMAG.a.1060-b74] Zhang, M., Guan, Z., Zhang, Y., Sun, W., Li, W., Hu, J., Li, B., Ye, G., Meng, H., Huang, X., Lin, X., Wang, J., Liu, J., Li, B., & Li, Y. (2022). Disrupted coupling between salience network segregation and glucose metabolism is associated with cognitive decline in Alzheimer’s disease—A simultaneous resting-state FDG-PET/fMRI study. NeuroImage. Clinical, 34, 102977. 10.1016/j.nicl.2022.102977 35259618 PMC8904621

[IMAG.a.1060-b75] Zhong, X., Van Lankveld, H., Mathew, A., & Chen, J. J. (2025). The link between steady-state EEG and rs-fMRI metrics in healthy young adults: The effect of macrovascular correction. bioRxiv, 2025.06.06.658306. 10.1101/2025.06.06.658306 PMC12797145 41537053

[IMAG.a.1060-b76] Zhong, X. Z., & Chen, J. J. (2022). Resting-state functional magnetic resonance imaging signal variations in aging: The role of neural activity. Human Brain Mapping, 43(9), 2880–2897. 10.1002/hbm.25823 35293656 PMC9120570

[IMAG.a.1060-b77] Zhong, X. Z., Polimeni, J. R., & Chen, J. J. (2024). Predicting the macrovascular contribution to resting-state fMRI functional connectivity at 3 Tesla: A model-informed approach. Imaging Neuroscience, 2, 1–22. 10.1162/imag_a_00315 PMC12290778 40800337

[IMAG.a.1060-b78] Zhong, X. Z., Tong, Y., & Chen, J. J. (2024). Assessment of the macrovascular contribution to resting-state fMRI functional connectivity at 3 Tesla. Imaging Neuroscience, 2, 1–20. 10.1162/imag_a_00174 PMC12272202 40800526

