# Supplementary material for: The link to steady-state oxidative metabolism and hemodynamics varies across rs-fMRI metrics: A whole-brain assessment using macrovascular correction
Source: Imaging Neurosci (Camb). 2025 Dec 11;3:IMAG.a.1060. doi: 10.1162/IMAG.a.1060 (PMC13288501; doi:10.1162/IMAG.a.1060)
Supplement: Supplementary Material [file IMAG.a.1060_supp.pdf]

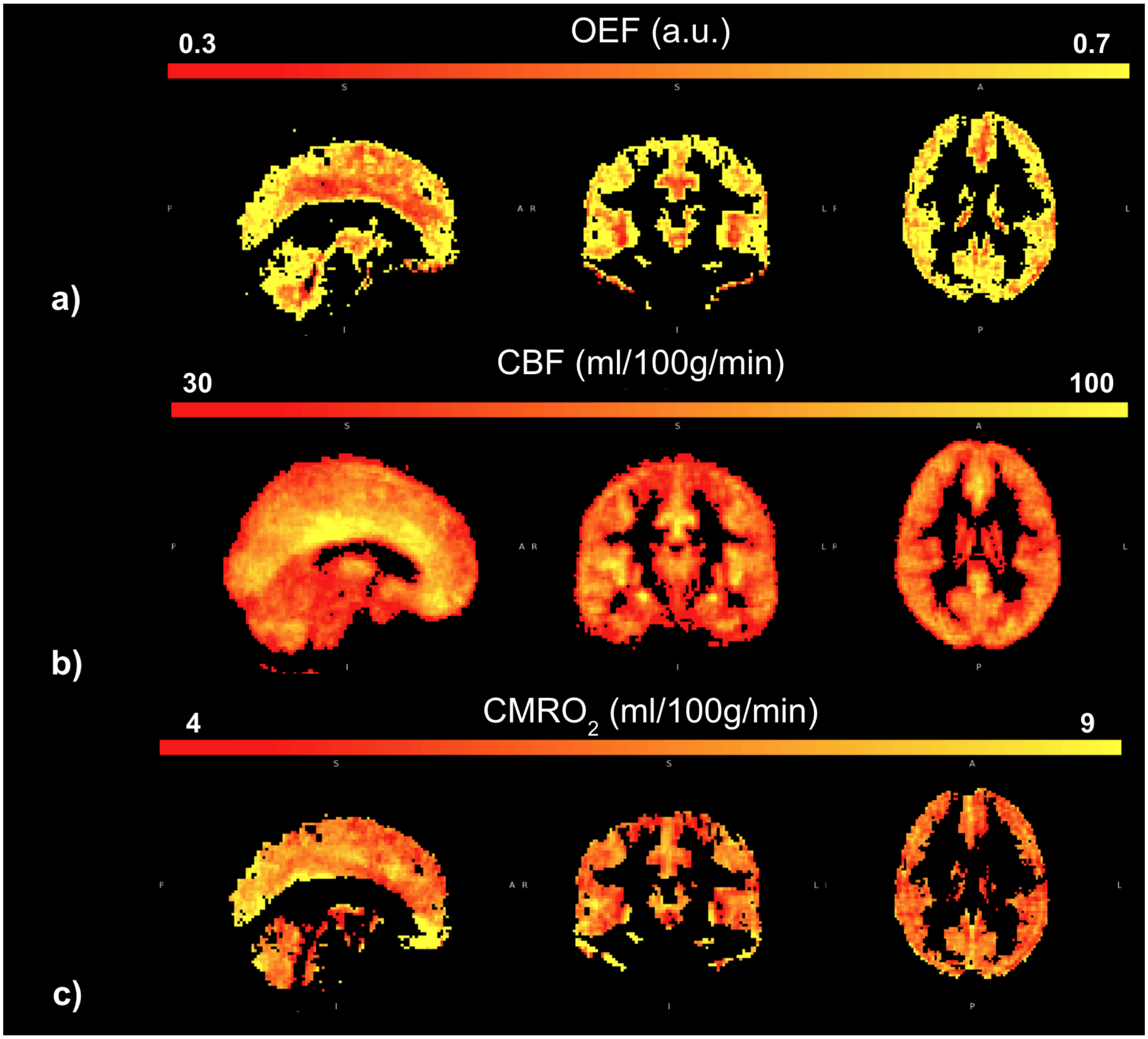

Figure S1. Baseline physiological variables across all participants in MNI 2mm space. a) OEF; b) CBF; c) CMRO$_2$. OEF and CMRO2 maps were masked based on OEF thresholds ranging from 0 to 1. Two voxels eroded the brain mask to remove the region with the coregistration error.

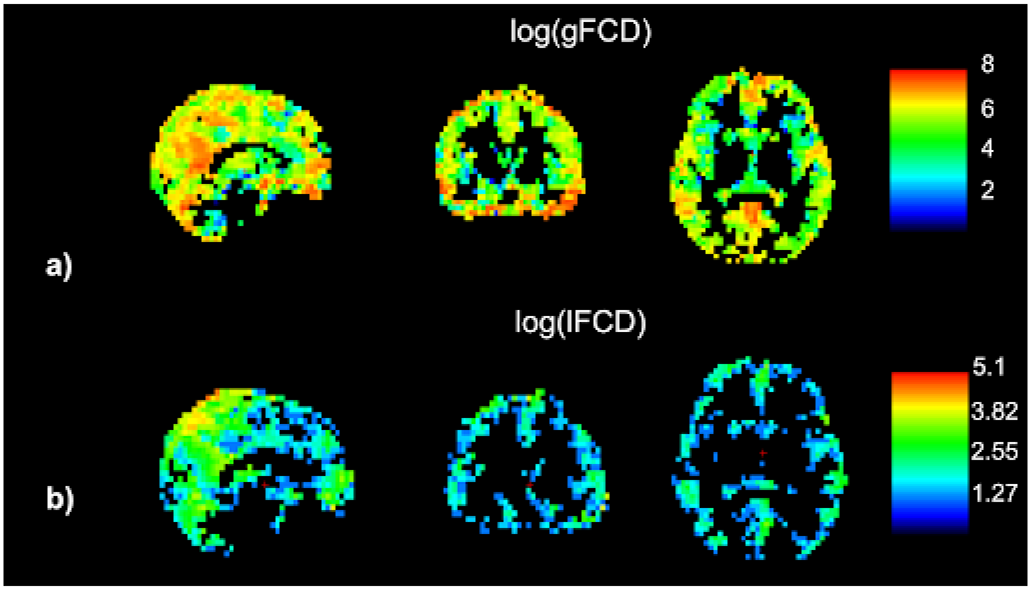

Figure S2. rs-fMRI metrics (network-independent) change after macrovascular correction (Data shown from a representative dataset). a) gFCD; b) lFCD.

Variations across networks

Network definitions varied depending on the rs-fMRI-based FC values, which were altered by macrovascular correction. This resulted in pre-correction and post-correction sets of network ROIs. **Figure S3** illustrates that there were no notable differences across networks in terms of $CMRO_2$, nor are there for OEF and CBF. There were no significant differences in rs-fMRI metrics across all seven networks (the differences were smaller than the intra-network standard deviation) for either seed-based or seed-independent metrics, despite the appearance of heterogeneity in the values across networks. The baseline physiological variables maps for the whole brain were provided in **Figure S1** and baseline gFCD and lFCD maps from a representative participant were provided in **Figure S2**.

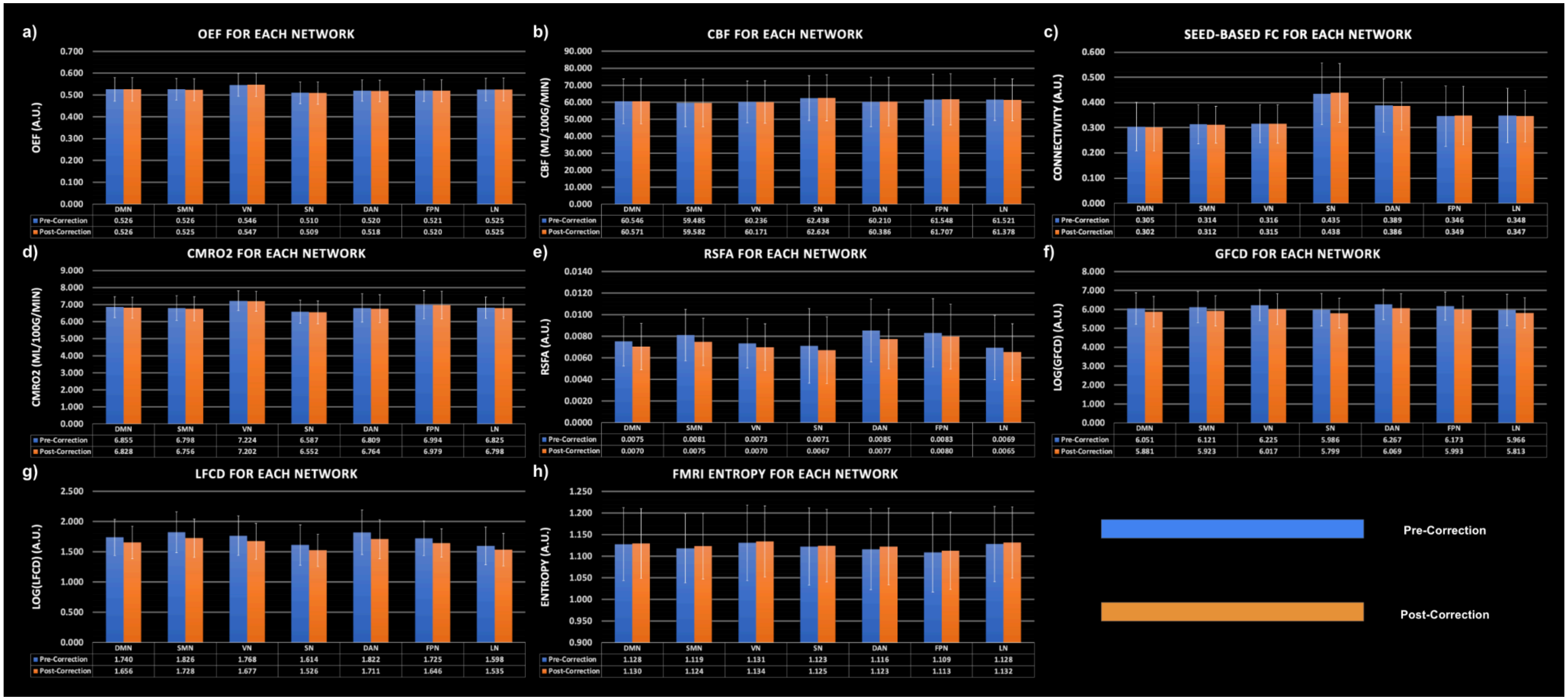

Figure S3. The average $CMRO_2$, OEF, CBF and rs-fMRI metrics using network ROIs are defined based on rs-fMRI data pre- and post-macrovascular correction. The rs-fMRI metrics corresponding to the post-correction ROIs are of course corrected for macrovascular contributions. Grouped bars from left to right: DMN, SMN, VN, SN, DAN, FPN, and LN. Blue: pre-macrovascular correction and orange: post-macrovascular correction. Error bars represent standard deviation.

Table S1. Comparison of the effect size from LME models with and without network IDs as random variables (post-correction). LME model with and without network IDs as random variables. Only associations with significant random effects are shown here. There is no noticeable difference between the effect size of significant effects before and after the network IDs are included as a random effect. Even the insignificant effects might go from positive to negative.

	Effect size for physiological variables (CBF, OEF, $CMRO_2$)		Effect size for sex interaction	
	With random effect	Without random effect	With random effect	Without random effect
lFCD vs CBF	0.41	0.37	0.13	0.11
lFCD vs OEF	-0.14	-0.09	-0.04	-0.01
Seed-based FC vs $CMRO_2$	0.05	-0.02	0.29	0.33
Seed-based FC vs CBF	0.22	0.25	0.23	0.25
Seed-based FC vs OEF	-0.31	-0.38	-0.13	-0.16

Venous signal simulation and correction

A whole-brain susceptibility map was generated numerically using the Fourier method (Eq. 1 and 2), in which a mask of the vasculature was constructed by upsampling macro-VANs to 0.175 mm isotropic resolution and zero-padding by the size of a full field-of-view on each side to avoid wraparounds resulting from cycle convolution (Cheng et al., 2009; Salomir et al., 2003). To calculate the susceptibility difference between blood and tissue outside macrovasculature, we assumed that the tissue type outside macrovasculature is grey matter (GM).

$$\Delta B_z = FT^{-1}[(\frac{1}{3} - \frac{k_z^2}{k^2})FT(\chi)\,] \quad (1)$$

$$\chi = \Delta\chi \cdot Hct \cdot (1 - Y) \quad (2)$$

FT denotes the Fourier transform, χ the local susceptibility, k_z the distance in the k space along the z-axis and k the distance in k-space ($k^2 = k_x^2 + k_y^2 + k_z^2$). R_2' is then calculated through the magnitude of the complex-valued mean magnetization of the dephasing spins resulting from the B_0 offset.

The mean BOLD signal was calculated as defined by Eq. 3 and 4.

$$S_{T2'} = |\mu(exp(i\gamma\Delta B_z TE))| \quad (3)$$

$$S = sin(\alpha)(1 - exp(-TR/T1))/(1 - cos(\alpha)exp(-TR/T1)\, exp(-TE/T2))S_{T2'} \quad (4)$$

where γ is the gyromagnetic ratio, and the operator $|\mu(\cdot)|$ represents the magnitude of the mean of a complex number. A summary of the values and definitions used for simulation parameters is provided in **Table S2**.

The simulations were conducted using our servers equipped with 14 cores of Intel Xeon X5687 CPU (at 3.6 GHz) (Intel Corporation, Santa Clara, CA, United States) and 180 GB of memory running Red Hat Enterprise Linux Server 7.7 (Red Hat Inc., Raleigh, NC, United States). A customized simulation script was written in MatLab 2019b (MathWorks Inc., Natick, MA, United States.).

Table S2. Simulation parameters and values. For all three simulation models, these values were set to default unless otherwise stated.

Parameter	Definition	Simulated Value	Source
$\Delta\chi$	Susceptibility of blood with fully deoxygenated blood	$4 \times \pi \times 0.27 \times 10^{-6}$	(Spees et al., 2001)
Hct	Hematocrit	0.4	Men: 40-54%; Women: 36-48% (Billett, 1990)
Voxel size	N/A	3.5 mm isotropic	According to in-vivo rs-fMRI acquisition protocol
TR	Repetition time	4.5 s	
TE	Echo time	30 ms	
α	Flip angle	90 deg	
B_0	Main magnetic field	3T	
Y_v	Venous oxygenation level	0.6	(Fan et al., 2014)
Y_{tissue}	Tissue oxygenation level	0.85	(Gagnon et al., 2015)

$T1_{blood}$	T1 of blood	1649 ms	(Zhang et al., 2013)
$T1_{tissue}$	T1 of tissue	1465 ms	(Shin et al., 2009)

Billett, H.H., 1990. Hemoglobin and Hematocrit, in: Walker, H.K., Hall, W.D., Hurst, J.W. (Eds.), Clinical Methods: The History, Physical, and Laboratory Examinations. Butterworths, Boston.

Cheng, Y.-C.N., Neelavalli, J., Haacke, E.M., 2009. Limitations of calculating field distributions and magnetic susceptibilities in MRI using a Fourier based method. Phys. Med. Biol. 54, 1169–1189. https://doi.org/10.1088/0031-9155/54/5/005

Fan, A.P., Bilgic, B., Gagnon, L., Witzel, T., Bhat, H., Rosen, B.R., Adalsteinsson, E., 2014. Quantitative oxygenation venography from MRI phase. Magn. Reson. Med. 72, 149–159. https://doi.org/10.1002/mrm.24918

Gagnon, L., Sakadžić, S., Lesage, F., Musacchia, J.J., Lefebvre, J., Fang, Q., Yücel, M.A., Evans, K.C., Mandeville, E.T., Cohen-Adad, J., Polimeni, J.R., Yaseen, M.A., Lo, E.H., Greve, D.N., Buxton, R.B., Dale, A.M., Devor, A., Boas, D.A., 2015. Quantifying the microvascular origin of BOLD-fMRI from first principles with two-photon microscopy and an oxygen-sensitive nanoprobe. J. Neurosci. 35, 3663–3675. https://doi.org/10.1523/JNEUROSCI.3555-14.2015

Salomir, R., de Senneville, B.D., Moonen, C.T.W., 2003. A fast calculation method for magnetic field inhomogeneity due to an arbitrary distribution of bulk susceptibility. Concepts Magn. Reson. 19B, 26–34. https://doi.org/10.1002/cmr.b.10083

Shin, W., Gu, H., Yang, Y., 2009. Fast high-resolution T1 mapping using inversion-recovery Look-Locker echo-planar imaging at steady state: optimization for accuracy and reliability. Magn. Reson. Med. 61, 899–906. https://doi.org/10.1002/mrm.21836

Spees, W.M., Yablonskiy, D.A., Oswood, M.C., Ackerman, J.J., 2001. Water proton MR properties of human blood at 1.5 Tesla: magnetic susceptibility, T(1), T(2), T*(2), and non-Lorentzian signal behavior. Magn. Reson. Med. 45, 533–542. https://doi.org/10.1002/mrm.1072

Zhang, X., Petersen, E.T., Ghariq, E., De Vis, J.B., Webb, A.G., Teeuwisse, W.M., Hendrikse, J., van Osch, M.J.P., 2013. In vivo blood T(1) measurements at 1.5 T, 3 T, and 7 T. Magn. Reson. Med. 70, 1082–1086. https://doi.org/10.1002/mrm.24550
